# Nutritional and Phytochemical Composition of Andean *Lupinus mutabilis* Sweet Germplasm from Ecuador

**DOI:** 10.3390/plants15132008

**Published:** 2026-06-29

**Authors:** Diego Rodríguez-Ortega, Iván Samaniego, José Luis Zambrano, Wilma Llerena-Silva, Leroy Lopez, Jhunior Marcía-Fuentes, Santiago Pereira-Lorenzo, Dani Ochoa-Cervantez

**Affiliations:** 1 International PhD Program in Agriculture and Environment for Development, University of Santiago de Compostela, 27002 Lugo, Spain; diego.rodriguez@iniap.gob.ec; 2 Programa de Leguminosas y Granos Andinos, Estación Experimental Santa Catalina, Instituto Nacional de Investigaciones Agropecuarias (INIAP), Cutuglahua 171107, Ecuador; 3 Departamento de Nutrición y Calidad, Estación Experimental Santa Catalina, Instituto Nacional de Investigaciones Agropecuarias (INIAP), Cutuglahua 171107, Ecuador; ivan.samaniego@iniap.gob.ec; 4 Postgraduate Program in Food Development and Innovation, University of the Americas (UDLA), Quito 170503, Ecuador; 5 Ingeniería en Agroempresas, Colegio de Ciencias e Ingenierías, Universidad San Francisco de Quito, Quito 170901, Ecuador; jlzambrano@usfq.edu.ec; 6 Faculty of Industry and Production Sciences, Food Engineering, State Technical University of Quevedo (UTEQ), Km 7 1/2 Via Quevedo-El Empalme, Los Ríos 120313, Ecuador; wllerenas@uteq.edu.ec; 7 Department Entomology, Texas A&M University, College Station, TX 77842-4002, USA; lrl1317@tamu.edu; 8 Faculty of Technological Sciences, Universidad Nacional de Agricultura, Catacamas 16201, Honduras; 9 Department of Crop Production and Engineering Projects, Campus Terra, University of Santiago de Compostela, 27002 Lugo, Spain; santiago.pereira.lorenzo@usc.es; 10 Dirección de Planificación y Desarrollo, Universidad Nacional de Ciencias Forestales, Colonia Las Américas, Siguatepeque 12111, Honduras; d.ochoa@unacifor.edu.hn

**Keywords:** *Lupinus albus*, *Lupinus angustifolius*, altramuz, chocho, lupin or tarwi

## Abstract

Lupinus is recognized as a nutrient-dense legume rich in protein, raw fiber, antioxidants, and unsaturated fatty acids, contributing significantly to human nutrition and health. In Ecuador, the Andean Crops and Plant Genetic Resources program of INIAP maintains a germplasm bank comprising 257 uncharacterized accessions. This study aimed to evaluate the nutritional and phytochemical composition of ten promising sweet *Lupin* genotypes (*L. mutabilis*) exhibiting good agronomic characteristics, resistance and/or tolerance to biotic and abiotic stresses, superior grain quality and significantly reduced seed alkaloid content in experimental trails. These genotypes were compared with two accessions of *L. albus* and *L. angustifolius* used as control genotypes. Except for carbohydrate content, *L. mutabilis* genotypes exhibited similar or superior nutritional profiles compared to genotype controls with high protein (44.7%), fat (19.91%), and ash (4.16%) contents and reduced alkaloid concentrations, notably, two genotypes LmAnds16 and LmFRs43 with 0.04%. However, it exhibited the highest polyphenol (8.84 mg·g^−1^) and flavonoid (0.67 mg·g^−1^) concentrations and antioxidant activity for ABTS (19.94 µmol TE·g^−1^) and FRAP (300.30 µmol TE·g^−1^) on a dry weight basis (DW). These results are important for the generation of new varieties of *Lupinus* focused on its nutritional quality and to produce nutraceutical and functional foods.

## 1. Introduction

*Lupinus mutabilis* Sweet is an Andean grain legume with deep historical roots in Ecuador, having constituted an integral component of indigenous dietary practices since the 17th century. Currently, it represents a fundamental element within crop rotation systems across the Ecuadorian Andean region, demonstrating cultivation viability even in arid and semiarid zones (250–1000 mm annual precipitation) at elevations ranging from 2700 to 3800 m.a.s.l. [1,2]. Beyond is exceptional adaptive capacity, this legume facilitates soil amelioration through biological atmospheric nitrogen fixation [3] and improves water infiltration [4]. It can remediate contaminated soils with heavy metals [3] and has the capacity to release Phosphorus (P) and Potassium (K), making them available for plant use [5]. Additionally, they are being used as green manure, making them ideal for sustainable production [4].

The genus Lupinus comprises nearly 300 species, but only four have been domesticated: *L. albus* (white lupin), *L. luteus* (yellow lupin), *L. angustifolius* (narrow-leaved or blue lupin), and *L. mutabilis* (Andean lupin) [4,6]. According to the FAO (2022) [7], worldwide Lupinus production reached 1,644,690 tonnes, led by Oceania (957,500 t) and Europe (570,432 t). Australia leads global production with 957,500 tonnes of *L. angustifolius*, *L. albus*, and *L. luteus*, followed by Poland (354,330 t) and Russia (105,544 t) [7]. In contrast to the other three species, *L. mutabilis* Sweet (also known as tarwi, chocho or Andean lupin) is primarily cultivated in the Andean region of South America [1,7,8].

The seeds of *L. mutabilis* are an important food source in Ecuador, Peru and Bolivia [4,9]. Peru leads production (1709 t) followed by Ecuador, which reported 1362 t in 2022, with lower production in Bolivia (420 t) according to its Ministry of Productive Development, although no official data have been available since 2017 [7].

Despite lupins being an ancestral food in Ecuador, their cultivation experienced a precipitous decline (94.4%) during the twentieth century, falling from 3116 to 174 t between 1970 and 1980, nearly disappearing from the national agro-productive landscape. In the southern provinces (Cañar, Azuay, and Loja), cultivation had practically been lost, with only isolated plants grown as ornamentals, whose growers were even unaware of their nutritional potential. Faced with the loss of agrodiversity, the National Institute of Agricultural Research of Ecuador (INIAP) implemented strategies for the rescue, characterization, and conservation of Lupinus species through its Legume Program. Systematic collections of national materials from Chimborazo and Imbabura were conducted, which, together with 21 accessions from Peru and Bolivia provided by the Institute of Nutrition in Lima, enabled the establishment of the Andean Crops and Plant Genetic Resources Sections. This effort resulted in the creation of a germplasm bank containing 257 accessions, whose catalog was published in 1998 [10,11].

Between 1998 and 2016, INIAP executed a comprehensive rescue and agro-industrial development program that enabled the agroecological zoning of the crop, with characterization of production systems in four provinces, encompassing even post-harvest management and agro-industrial processing. The work culminated in the release of two commercial varieties: INIAP 450 Andino (1999) [12], characterized by its earliness, white grain, and high yield potential, and INIAP 451 Guaranguito (2010) [1,13].

Since this period, INIAP has directed its efforts toward genetic improvement through hybridization, obtaining lupin materials that were differentiated using phenotypic markers such as flower color and are characterized by higher production yields, improved grain quality, and anthracnose resistance [14]. The rescue of ancestral crops undertaken by INIAP demonstrates the importance of technology transfer to the productive sector. Currently, 82% of lupin production is destined for sale, generating employment and income in poor rural communities throughout the agrifood chain (production, processing, and marketing).

Private companies and restaurants add value to this legume through ready-to-consume products. The remaining 18% of production contributes to reducing malnutrition in impoverished communities and forms part of school feeding programs. In Ecuador, Lupins are grown in seven provinces of the Ecuadorian highlands between 2700 and 3800 m above sea level. Two improved varieties are available: INIAP 451 Guaranguito and INIAP 450 Andino, the second being the most widely planted in the country’s production areas [14].

Lupins have gained increasing attention as nutrient-dense legumes, attributed to their remarkable nutritional composition characterized by being among the highest protein content documented in plant-based sources, exceeding that of other legumes such as peas (*Pisum sativum*), faba beans (*Vicia faba*) and common beans (*Phaseolus vulgaris*) [15,16]. Recent studies suggest that lupin seeds have potential as a healthy food with multiple applications for a wide range of consumers, including vegetarians and individuals with diabetes or gluten intolerance. Due to their high content of essential amino acids such as lysine, they could be combined with cereal-based diets (rich in cysteine and methionine), thereby improving the protein quality of vegetarian diets [15,17].

Furthermore, due to their abundance of essential macronutrients, Lupins are rich in bioactive compounds, including flavonoids, phytosterols, tocopherols and triterpenes [18,19,20]. Interestingly, processed flours (debittered, spray-dried, extruded) of *L. mutabilis* still present valuable levels of these bioactive compounds [18]. Their therapeutic promise is attributed to their antioxidant, anti-inflammatory, antibacterial and anti-carcinogenic, anti-obesity and hypoglycemic properties [17,20,21].

In addition to their nutritional benefits, Lupinus plants contain toxic substances such as quinolizidine alkaloids (QAs), secondary metabolites frequently found in the Leguminosae family that form part of plant defense systems against natural enemies such as insect pests and predators. In seeds, QAs impart bitter taste, limiting their food use [19,22]. Commercial varieties cultivated in Ecuador still maintain high QA levels, requiring elimination processes that, depending on the genotype, result in partial removal of bitterness. Despite the high content of quinolizidine alkaloids, the seeds have a high nutritional value [19,23]. All improved and native varieties grown in Ecuador are bitter (high alkaloid content) [18,19,24]. Traditional debittering methods based on prolonged soaking and washing remain widely used in the Andean region [25], while more recent technologies have improved efficiency and reduced processing time [24]. An alternative strategy is the development of genetically low-alkaloid (“sweet”) germplasm through breeding programs [14]. These materials offer important advantages by reducing or eliminating the need for post-harvest debittering; however, lower alkaloid concentrations may also reduce the natural chemical defense of plants against certain pests and pathogens. Therefore, the identification and evaluation of low-alkaloid genotypes with adequate agronomic performance remain a major objective in lupin breeding programs.

The INIAP Andean Legume and Grain Improvement Program has developed improved germplasm of the four Lupinus species, which exhibits significantly reduced alkaloid levels [26]. The plants and seeds have been characterized morphologically and agronomically. However, biochemical and nutritional composition remains undetermined. Despite these programs, housing comprehensive germplasm from breeding programs in Ecuador remains lacking, despite these programs housing comprehensive germplasm collections with potential for selecting genotypes that exhibit a superior nutritional profile characteristic [2,14,24].

Consequently, this study aimed to determine the nutritional and phytochemical composition of ten promising sweet and bitter commercial and breeding germplasm accessions of *L. mutabilis*, generating valuable information for breeding programs in the Andean region and the agro-industrial sector.

## 2. Results and Discussion

### 2.1. Proximal Composition

#### 2.1.1. Protein

The protein content of *Lupinus mutabilis* accessions belonging to the INIAP germplasm bank showed high values ranging from 40.25 to 47.17% (Table 1). The commercial variety INIAP 459 Andino recorded the highest protein content (47.17%), differing statistically (*p* < 0.05) from the rest of the materials evaluated. However, other improved genotypes such as LmProin17 (46.27%), LmFR11s67 (45.86%), and LmAnds16 (44.89%) had protein levels comparable to the commercial variety INIAP 451 Guaranguito, forming statistically heterogeneous groups. In contrast, the genotypes LmProin19 (40.25%), LmAnds167 (41.59%), LmFR9s43 (42.13%), and LmFR11 (43.19%) showed significantly lower protein contents compared to other accessions of the same ecotype.

These results show that protein composition can vary depending on genetic origin and genotypic adaptability to soil and climate conditions, which generates a marked differentiation between accessions from the same geographical area and subject to homogeneous agronomic management. These results are consistent with ranges reported for *L. mutabilis* by Boukid and Pasqualone [27], Briceño-Berru et al. [8] and Carvajal-Larenas et al. [25] which reported protein contents ranging from 29.93 to 60%.

The average protein content of *Lupinus mutabilis* (44.74%) was significantly higher than the values observed in the genotypes introduced, used as controls: *Lupinus angustifolius* (Lang4318, 28.58%) and *Lupinus albus* (Lalb2742, 34.29%). These findings are consistent with previous reports indicating that pearl lupin (43.3%) and yellow lupin (42.2%) seeds have higher protein contents than white lupin (38.2%) and narrow-leaved blue lupin (33.9%). The lower protein composition observed in the introduced germplasm, compared to the values reported in the literature, suggests a modulating effect of local environmental factors (altitude, soil characteristics, and climatic conditions) [2]. The results confirm that genotypes are the determining factor in grain protein composition, while growing conditions have a modulating effect on these characteristics [15].

A high protein content was observed in the materials developed by INIAP positions; these *L. mutabilis* genotypes are a plant protein source of high nutritional quality, with the potential to constitute a sustainable alternative to animal proteins in human nutrition.

#### 2.1.2. Total Fat

The lipid content (Table 1) of *Lupinus mutabilis* accessions exhibited a different pattern of variation than that observed for protein, evidencing an effect of parental genotype on materials derived from crosses. The commercial variety INIAP 451 Guaranguito recorded the highest fat content (23.87%). The accessions LmAnds16, LmAnds77, and LmAnds167 had similar lipid contents (18.53–18.87%), attributable to the inheritance of one of their genetic parents (INIAP 450 Andino). However, the hybridization process reduced their lipid concentration compared to the parental INIAP 450 Andino (20.11%). In the pink-flowered accessions, no marked predominance of the parental phenotype associated with flower color was observed, with considerable variability in fat content: LmFR9s43 (18.45%), LmFR11 (19.27%), and LmFR11s67 (21.40%).

The lines generated by INIAP through crosses between genotypes with low alkaloid content (0.04%) and high alkaloid content (1.5–2.0%) had higher lipid contents than the previously adapted Bolivian accessions (PROINPA donation). The accession LmProin19 exhibited the lowest fat content (16.03%) among all genotypes evaluated, while LmProin17 (18.32%) showed values comparable to other accessions studied. These results are consistent with those reported by Sotelo-Méndez et al. [28] and Johnson et al. [20], with fat contents ranging from 18 to 21.16%. Despite the wide intraspecific variability observed in *L. mutabilis*, the average lipid content (19.91%) of this species was significantly higher than that of the control genotypes: *Lupinus angustifolius* (Lang4318) and *Lupinus albus* (Lalb2742), were 8.07 and 12.07%, respectively. There are findings that have already been reported by several authors [20,27,29].

These results suggest that the chemical composition of the grain can be modulated by soil and climate factors associated with the growing area, which can also influence morphological characteristics such as plant height and branching [2,15].

#### 2.1.3. Fiber

Fiber content showed a marked effect of the parental genotype in each of the accessions evaluated. The *Lupinus mutabilis* genotypes derived from the parental INIAP 450 Andino had fiber contents between 13.08 and 15.50%, with similarities observed between the LmAnds16 and LmAnds167 materials, while LmAnds77 exhibited a significantly lower value. In pink-flowering materials, the fiber content was more consistent: 14.10% (LmFR9s43), 14.37% (LmFR11), and 14.68% (LmFR11s67). The accessions introduced from Bolivia had the lowest fiber contents: 12.56% (LmProin19) and 12.62% (LmProin17), typical of PROINPA materials.

Genetic diversity emerged as a substantial contributor to the observed variation, with *L. mutabilis* genotypes exhibiting differences in fiber content despite being cultivated under uniform environmental conditions. Among the materials developed by INIAP, the commercial variety INIAP 451 Guaranguito (15.50%) and accession LmAds167 (15.50%) had the highest fiber content among all *L. mutabilis* genotypes, exceeding the overall average (14.16%). The *Lupinus angustifolius* control accession (16.19%) was characterized by a higher fiber content than the *L. albus* (8.86%) and *L. mutabilis* materials, demonstrating considerable variability among domesticated lupine species.

Carvajal-Larenas et al. [25] and Abraham et al. [29] reported fiber content values of 6.2–11.0% for *L. mutabilis*. These values were 2 to 4% lower than those observed in the present study, suggesting that crude fiber composition may be influenced by edaphoclimatic conditions. The favorable cultivation conditions in Ecuador, particularly soil and climate, may have contributed to the higher fiber content observed. However, the fiber content values obtained for *L. albus* (8.86%) and *L. angustifolius* (16.19%) were consistent with those reported by Carvajal-Larenas et al. [25] that aligned closely to 8.9 and 16.0%, respectively. The prokinetic effect of dietary fiber is that it positively promotes a series of physiological functions, such as lowering blood cholesterol levels and controlling blood glucose levels [30].

#### 2.1.4. Carbohydrates

Based on the results presented in Table 1, Lupinus mutabilis accessions show wide variability in carbohydrate content (12.13 to 27.19%); however, these values are lower than those reported in samples of *Lupinus albus* (40.81%) and *Lupinus angustifolius* (42.22%). This indicates that carbohydrate content is a differential nutritional marker between species; however, the genotypes of origin of each material can influence carbohydrate accumulation, even when grown under the same conditions.

Therefore, no effect was observed from the crossing pattern (LmAnds 16, LmAnds77, LmAnds167, LmFR11, LmFR11s67, and LmFR9s43), improved commercial varieties (INIAP 450 Andino and INIAP 451 Guaranguito), and introduced germplasm (LmProin17 and LmProin19). According to Carvajal-Larenas et al. [31] the wide variability in carbohydrate contents is associated with genetic and agronomic factors, as *L. mutabilis* presents a high genetic variability due to its adaptation to microenvironments and natural selection. This variability has been particularly observed in plant shape, vegetative growth, susceptibility to frost and diseases, and macronutrients contents. Another study on *L. mutabilis*, by Briceño-Berru et al. [8] and Carvajal-Larenas et al. [31], reported carbohydrate contents for *L. mutabilis* ranging from 24.85% and 43.2%, showing a significant difference, almost double the average carbohydrate contents among *L mutabilis* genotypes (17.04%).

#### 2.1.5. Ash

No marked effect of parental genotype was observed in mineral content (ash), with values between 3.13 and 6.02 identified for the *Lupinus mutabilis* genotype. The accession LmAnds16 presented the highest ash content (6.05%), followed by the accessions LmFr9s43 (4.99%), LmAnds77 (4.88%), and LmAnds167 (4.86%). The accessions LmProin17 (4.39%) and LmFRs67 (4.29%) formed a different group. The samples of *L. mutabilis* INIAP 451 Guaranguito (4.02%) and LmProin19 (3.99%) showed statistically similar values to the samples of *Lupinus albus* (3.98%) and *Lupinus angustifolius* (3.95%). However, samples of the commercial variety INIAP 450 Andino (3.28%) and the improved genotype LmFR11 (3.13%) were characterized by low mineral content, associated with their low fiber content. These results corroborate previous findings by different authors [8,15,27,31].

Considering their nutritional composition, it can be confirmed that *Lupinus mutabilis* seeds are a source of plant-based protein with a balanced content of amino acids and storage proteins such as albumins or globulins, non-digestible carbohydrates, and fiber, as well as having a high fat content with a notable proportion of unsaturated fatty acids. This combination of nutrients makes them a potential substitute for animal proteins such as meat, eggs, cheese, and others. This makes it a viable alternative for promoting food security and agricultural sustainability in developing countries with low availability and accessibility to a balanced diet [15,32].

#### 2.1.6. Moisture

Moisture content is a critical quality and stability parameter in proximal composition analysis, as it directly influences the physicochemical, microbiological, and storage behavior of seeds and seed-derived products. From an analytical standpoint, moisture content represents the baseline against which all other proximate fractions (protein, fat, fiber, carbohydrates, ash) are expressed on a dry-matter basis; consequently, variation in moisture among accessions can partially influence the apparent concentration of other nutrients if not properly standardized, underscoring the importance of reporting both fresh-weight and dry-matter values in comparative germplasm studies.

From a food safety and post-harvest perspective, moisture content is a key determinant of grain storability and shelf-life, since seeds with higher moisture levels (>10–12%) are generally more susceptible to enzymatic degradation, lipid oxidation, and microbial (fungal and bacterial) proliferation during storage [33]. In this study, all *L. mutabilis* accessions exhibited moisture values below 9%, which is consistent with safe storage thresholds commonly recommended for legume and pseudocereal grains, and suggests adequate post-harvest drying and conditioning of the evaluated germplasm [33,34].

The relatively narrow range observed among *L. mutabilis* genotypes (6.32–8.73%) further indicates that moisture content in this species is influenced more by genotype-dependent seed coat characteristics and tegument permeability than by environmental variability alone, although confirmation of this would require multi-environment trials.

Moisture content differed significantly among the evaluated genotypes (*p* < 0.05), ranging from 6.32% in LmAnds77 to 9.21% in the control accession ECU 4318 (Table 1). The commercial cultivars INIAP 450 Andino and INIAP 451 Guaranguito exhibited moisture contents of 8.51% and 6.55%, respectively. Overall, the evaluated germplasm presented low moisture levels (mean = 7.60%; CV = 2.05%), indicating adequate seed drying and storage conditions [25,33,34].

### 2.2. Fatty Acid Profile

In the *Lupinus* spp. cultivars studied, a medium- to long-chain fatty acid profile (C16 to C22) characteristic of oilseeds was identified; this is because their enzyme systems lack specific metabolic pathways for producing short-chain (C4 to C12) or very long-chain (>C24) fatty acids. The fatty acid profile consisted of saturated fatty acids (SFAs), monounsaturated fatty acids (MUFAs), and polyunsaturated fatty acids (PUFAs), whose concentrations varied significantly depending on the species and genotype [35], as detailed in Table 2.

#### 2.2.1. Saturated Fatty Acids

The SFA content (Table 2) consisted of palmitic acid (C16:0), stearic acid (C18:0), and behenic acid (C22:0), which is expected to be minimal due to its health implications. Among the SFA, palmitic acid is the one found in the highest proportions; however, its levels are lower than MUFA and PUFA. The *L. mutabilis* genotype showed an average of 10.66% palmitic acid and smaller amounts were found for stearic acid, with an average of 6.83%, and behenic acid, with an average of 0.84%. Sotelo-Méndez et al. [28] and Carvajal-Larenas et al., [31] report for *L. mutabilis* ranges of 8.83% and 13.9% for palmitic acid, 2.0% to 8.6% for stearic acid and 0.6% to 0.9% for behenic acid, values that are consistent with those found in this study. These results were comparable to the values reported by Sotelo-Méndez et al. [28] and Carvajal-Larenas et al., [31], who identified palmitic acid (8.83 and 13.9%), stearic acid (2.0 to 8.6%), and behenic acid (0.6 to 0.9%) in *L. mutabilis* genotypes.

Despite this, the average SFA content in Lupinus spp. cultivars shows statistically significant differences between genotypes and control samples. The palmitic, stearic, and behenic acid contents of *L. albus* and *L. angustifolius* were within the range reported for all *L. mutabilis* genotypes from the germplasm bank developed and improved by INIAP. In the control samples, *L. angustifolius* had the highest percentages of palmitic acid (12.31%) and stearic acid (7.07%) compared to *L. albus*, which had concentrations of 8.0% and 2.37% for C16:0 and C18:0, respectively. In contrast, behenic acid was higher in *L. albus* (3.03%) than in *L. angustifolius* (1.77%).

The sweet lupine (*L. mutabilis*): LmAnds16, LmAnds77, LmAnds167, LmFR11, LmFR9s43, INIAP 450 Andino, INIAP 451 Guaranguito, LmProin17 and LmProin19 had palmitic acid contents close to the values reported for *L. angustifolius*, ranging between 10.16 and 13.57%; however, the LmFR11s67 genotype (8.46%) had values closer to those of *L. albus*. In terms of stearic acid content, the sweet lupine genotypes had higher percentages (8.81 to 9.91%) in LmAnds16, LmAnds77, and LmFR11s67, whose values were higher than other genotypes of *L mutabilis*, *L. albus*, and *L. angustifolius*. The commercial varieties from the INIAP 450 Andino (5.57%) and INIAP 451 Guaranguito (7.39%) germplasm banks had a similar average content to *L. angustifolius* seeds, with statistical similarities to Guaranguito seeds.

The samples of LmAnds167, LmFR11, LmFR9s43, LmProin17, and LmProin19 had the lowest C18:0 values (4.45 to 6.01%), among the *L. mutabilis* genotypes studied; these were higher than the *L. albus* samples, which had the lowest value among all the germplasm studied. Sweet lupine (*L. mutabilis*) materials were characterized by their low behenic acid content (0.05 to 1.19%), differing from the cultivars *L. angustifolius* (1.77%) and *L. albus* (3.03%).

#### 2.2.2. Monounsaturated Fatty Acids

*Lupinus* spp. cultivars showed variability in monounsaturated fatty acid content (Table 2), with marked differences between the genotypes *L. albus* (Lalb2742), *L. mutabilis*, and *L. angustifolius* (Lang4318). *L. albus* seeds had the highest oleic acid content (66.70%), with no statistically comparable lupine genotypes. The oleic acid levels identified in this cultivar were comparable to olive 22 cultivars from Antakya, Türkiye, where values between 67.20 and 77.50% were identified [35].

The *L. mutabilis* samples showed wide variability in oleic acid concentrations, with superior quality grains ranging from 57% (LmAnd577) to 59% (LmFR11s67 and LmProin19), followed by seeds with an average content of 49.85 to 53.51% (LmAnd5167, LmAnd516, LmProin17, and LmFR9:43). The commercial samples INIAP 451 Guaranguito (48.7%), INIAP 450 Andino (45.33%), and LmFR11 (45.61%) had lower levels of oleic acid than the sweet lupin cultivars. However, these values were higher than the *L. angustifolius* samples, which showed a very low MUFA percentage of 38.34%.

These results are consistent with those reported by Carvajal-Larenas et al. [25], who found oleic acid as the predominant MUFA component in *L. albus* (54.0%) and *L. angustifolius* (33.9%) germplasm. According to Toplu et al. [35], geographical and environmental conditions, particularly temperature and genotype environment interactions, influence lipid profile and oil quality. Oleic acid (C18:1n9C) concentration in cultivars depends on genotype adaptation and the physiological and biochemical responses to environmental conditions. This would explain the observed behavior of improved sweet lupin genotypes, which did not exhibit a marked effect of their genetic parentage.

#### 2.2.3. Polyunsaturated Fatty Acids

Polyunsaturated fatty acid content exhibited an inverse pattern to that observed for MUFAs, with *L. angustifolius* cultivars showing higher PUFA concentrations than *L. mutabilis* and *L. albus* germplasm. In general, lupin seeds presented elevated omega-6 and omega-3 percentages, with the highest concentration observed in accession Lang4318 of *L. angustifolius* (38.29%). *L. mutabilis* germplasm exhibited substantial variability in PUFA content, with accession LmFR11 (35.77%) comparable to the *L. angustifolius* control, followed by commercial variety INIAP 450 Andino (34.45%), LmAnds167 (34.15%), INIAP 451 Guaranguito (31.90%), LmProin17 (28.54%), LmFR9s43 (28.37%), LmProin19 (24.83%), LmAnds16 (24.64%), LmFR11s67 (20.75%), and LmAnds77 (18.03%). The *L. albus* control genotype Lalb2742 exhibited the lowest omega-6 and omega-3 content (15.46%).

Regarding polyunsaturated fatty acids (Table 2), linoleic acid was predominant. *L. mutabilis* genotypes exhibited linoleic acid contents ranging from 16.38% (LmAnds77) to 33.07% (LmFR11), with a mean of 25.63%. Three *L. mutabilis* genotypes exceeded 30%: LmFR11 (33.07%), INIAP 450 Andino (31.71%) and LmAnds167 (31.63%). Linolenic acid levels in the evaluated germplasm were relatively low, averaging 2.52% for *L. mutabilis*, 4.60% for *L. albus* and 6.00% for *L. angustifolius.* Lang4318 (*L. angustifolius*) exhibited the highest linoleic acid content (32.29%).

These findings are consistent with previous studies reporting linoleic acid contents of 23.63–39.60% and linolenic acid contents of 1.9–3.0% for *L. mutabilis* [25]. Similarly, Carvajal-Larenas et al. [31] reported linoleic acid contents of 18.7% and 40.3%, and linolenic acid contents of 8.6% and 5.6% for *L. albus* and *L. angustifolius*, respectively, which align with the present results (Table 2). The elevated linoleic acid concentrations observed in *L. angustifolius* and *L. mutabilis* corroborates previous findings.

Monounsaturated fatty acids (MUFAs) and polyunsaturated fatty acids (PUFAs) prevent, and regenerate cellular damage induced by saturated fatty acids (SFAs), making them useful for the prevention and supportive therapy of metabolic diseases such as type 2 diabetes. Oleic acid (C18:1n9C), an unsaturated fatty acid, has been reported to reduce the risk of cardiovascular disease when consumed over the long term [15,20,28,36,37].

### 2.3. Minerals

Mineral compositions analysis (Table 3) revealed significant differences among Lupinus genotypes across all macroelements and microelements (ANOVA, Tukey’s test, *p* < 0.05). LmProin19 exhibited the highest concentrations of Copper (Cu: 8.47 ppm), Magnesium (Mg: 2.51 g∙kg^−1^), Sodium (Na: 0.34 g∙kg^−1^) and Phosphorus (P: 8.20 g∙kg^−1^). Conversely, the lowest concentrations of Cu were observed in LmFR9s43 (4.03 ppm) and control of *L. albus* (Lalb2742: 4.07 ppm), while Mg contents were minimal in Lalb2742 (1.86 g∙kg^−1^) and LmFR11 (1.85 g∙kg^−1^). Sodium levels were particularly low in LmAnds77 (0.06 g∙kg^−1^) and INIAP 451 Guaranguito (0.07 g∙kg^−1^), with LmFR11 displaying the lowest Phosphorus content (P: 2.99 g∙kg^−1^).

With respect to microelements, *L. angustifolius* (Lang4318: 62.24 ppm) and INIAP 451 Guaranguito (59.78 ppm) demonstrated the highest Iron (Fe) concentrations, whereas LmFR9s43 exhibited the lowest value (33.87 ppm). Calcium (Ca), levels were markedly elevated in Lang4318 (2.91 g∙kg^−1^) compared with LmAnds167 (0.83 g∙kg^−1^). Notably, *L. albus* (Lalb2742) displayed exceptional Manganese (Mn) accumulation (142.11 ppm), significantly exceeding other *Lupinus* genotypes, while LmFR9s43 showed the lowest content (20.14 ppm). The highest Zinc (Zn) and Potassium (K) concentrations were recorded in INIAP 451 Guaranguito (60.81 ppm) and in LmAnds16 (24.48 g∙kg^−1^), respectively, whereas LmFR9s43 and INIAP 450 Andino exhibited the lowest levels with 39.91 ppm of and 7.97 g∙kg^−1^, respectively.

Micronutrient profiling of *L. mutabilis* germplasm revealed considerable variability, with Zinc (Zn) and Iron (Fe) being particularly abundant, ranging from 31.91 ppm (LmFR9s43) to 60.81 ppm (INIAP 451 Guaranguito) and from 33.87 ppm (LmProin19) to 59.78 ppm (INIAP 451 Guaranguito), respectively. Manganese (Mn) and Copper (Cu) concentrations ranged from 20.14 ppm (LmFR9s43) to 33.77 ppm (LmProin19) and 4.03 ppm (LmFR9s43) to 8.47 (LmProin19) ppm, respectively, exhibiting more limited variation than other microelements.

Comparative analysis across Lupinus species revealed that *L. angustifolius* (Lang4318) achieved the highest Iron contents (62.25 ppm), substantially exceeding the *L. mutabilis* average (44.22 ppm), although INIAP 451 Guaranguito (59.78 ppm) approached this maximum. Similarly, Zinc concentrations were higher in *L. mutabilis* mean (45.17 ppm) with INIAP 451 Guaranguito again reaching 60.81 ppm. Notably, *L. albus* (Lalb2742) demonstrated Manganese concentrations 3–7 times higher than other genotypes (139.78 and 144.45 ppm), establishing a distinct physiological signature across Lupinus species.

The micronutrient ranges obtained for *L. mutabilis* germplasm (Fe: 33.71–60.07 ppm; Cu: 3.99–8.89 ppm; Mn: 19.57–34.01 ppm; Zn: 39.40–61.34 ppm) were consistent with those previously reported by Ruiz-López et al. [21] (Fe 46.67–88.88 ppm, Cu 5.0–10.67 ppm, Mn 20.67–47.67 ppm, Zn 35.67–52.67 ppm) and Grela et al. [38] (Fe 56.63 ppm, Cu 7.21 ppm, Mn 49.11 ppm, Zn 52.71 ppm), supporting the nutritional quality of this germplasm for breeding and agronomic applications.

Macronutrients composition of *L. mutabilis* germplasm demonstrated considerable genetic variation (Table 1). Potassium (K) exhibited the widest range 10.53 g∙kg^−1^ (INIAP 451 Guaranguito) to 24.48 g∙kg^−1^ (LmAnds16). Sodium (Na) contents were notably low and restricted (0.06 g∙kg^−1^ to 0.34 g∙kg^−1^), with minimal accumulation across all evaluated genotypes (LmAnds77 and LmFR9s43). Calcium (Ca) levels ranged from 0.83 to 1.66 g∙kg^−1^ (LmAnds167 and LmProin19, respectively), while Magnesium (Mg) concentrations varied between 1.85 and 2.51 g∙kg^−1^ (LmFR11 and LmProin19). Phosphorus (P) contents spanned 4.06 g∙kg^−1^ (LmFR9s43) and 8.20 g∙kg^−1^ (LmProin19), reflecting substantial genotype-dependent variation. A comparative analysis with previously published data by Grela et al. [37] revealed that the macronutrient range obtained in this study was generally consistent with the reported average for *L. mutabilis* Potassium (K: 11.69 g∙kg^−1^), Sodium (Na: 0.15 g∙kg^−1^), Phosphorus (P: 5.79 g∙kg^−1^), Calcium (Ca: 1.28 g∙kg^−1^) and Magnesium (Mg: 2.17 g∙kg^−1^) all fell within the observed ranges. This concordance detected across micro- and macronutrient contents among *Lupinus* germplasm underscores the agronomic potential for identifying biofortified cultivars of *L. mutabilis* suitable for enhanced nutritional quality in breeding programs [16,39].

### 2.4. Functional Properties

The antioxidant profiles of the *Lupinus* germplasm collection are presented in Table 4. Genotype effects on antioxidant constitutes were confirmed through between seven and ten different significance levels (different letters) for the four variables analyzed.

Total polyphenol content concentrations displayed substantial genotype variation ranging from 2.20 to 15.6 mg GAE∙g^−1^. The elite accession INIAP 450 Andino achieved the maximum polyphenol accumulation at 15.6 mg GAE∙g^−1^, higher than the control *L. albus* (Lalb2742: 2.20 mg GAE∙g^−1^). This was followed by INIAP 451 Guaranguito (14.04 mg GAE∙g^−1^) and LmFR11 (13. 11 mg GAE∙g^−1^). In contrast, the control accessions *L. albus* (Lalb2742) and *L. angustifolius* (Lang4318) presented the lowest contents with 2.20 and 3. 11 mg GAE∙g^−1^, respectively.

Flavonoid accumulation ranged closely between 0.35 and 0.84 mg CE∙g^−1^, representing less pronounced genotypic differentiation compared to polyphenol content. The commercial cultivars INIAP 450 Andino (0.84 mg CE∙g^−1^), INIAP 451 Guaranguito (0.78 mg CE∙g^−1^) and LmFR11 (0.77 mg CE∙g^−1^) consistently exhibited the highest flavonoid contents, while control accessions demonstrated reduced accumulation *L. albus* (Lalb2742) and *L. angustifolius* (Lang4318), and LmProin19 (*L. mutabilis*) presented the lowest contents with 11.43, 12.69 and 13.80 mg CE∙g^−1^.

These results suggest that flavonoid biosynthesis is less responsive to genotypic variation in this *Lupinus collection*. FRAP analysis revealed pronounced genotypic differentiation in electron-donating capacity, ranging from 11.43 to 28.39 µmol TE·g^−1^. The highest antioxidant activity was observed in commercial varieties INIAP 450 Andino (28.39 µmol TE·g^−1^), followed by INIAP 451 Guaranguito (26.53 µmol TE·g^−1^) and germplasm LmFR11 (25.73 µmol TE·g^−1^).

These three accessions showed FRAP values superior to the control seeds, with *L. albus* (Lalb2742) and *L. angustifolius* (Lang4318) displaying the lowest reducing capacity at 11.43 and 12.69 µmol TE·g^−1^, respectively. The performance of LmProin19 (*L. mutabilis*) antioxidant activity (13.80 µmol TE·g^−1^) was intermediate between elite and control genotypes.

The ABTS antioxidant capacity demonstrated the greatest genotypic differentiation among all parameters evaluated, ranging from 38.95 to 432.98 µmol TE·g^−1^, the variation reflecting substantial intraspecific heterogeneity. The elite accession of INIAP 450 Andino exhibited superior performance at 432.98 µmol TE·g^−1^, followed by INIAP 451 Guaranguito (404.36 µmol TE·g^−1^), LmFR11 (393.96 µmol TE·g^−1^), and LmFR11s67 (381.51 µmol TE·g^−1^). The control accessions *L. albus* (Lalb2742: 38.95 µmol TE·g^−1^) and *L. angustifolius* (Lang4318: 1.48 µmol TE·g^−1,^) exhibited the lowest antioxidant capacity values. This pronounced variation underscores the substantial genotypic differentiation available for selection in breeding programs targeting enhanced antioxidant profiles. Among phenolic secondary metabolites of the *Lupinus* cultivar and plants, flavonoids represent bioactive compounds characterized by potent antioxidant activity, capable of neutralizing free radicals and thereby conferring protection against oxidative stress-related diseases, including cardiovascular dysfunction and type 2 diabetes mellitus [15,17,24].

The reference germplasm *L. mutabilis* demonstrated superior phytochemical composition relative to control seeds: *L. albus* and *L. angustifolius*. Total polyphenol (8.84 mg GAE·g^−1^), flavonoid (0.67 mg CE·g^−1^), FRAP (19.94 µmol TE·g^−1^) and ABTS (300.30 µmol TE·g^−1^) content in sweet Lupinus (*L. mutabilis*) substantially exceeded those of the control accessions *L. albus* (Lalb2742: 2.20 mg GAE·g^−1^; 0.46 mg CE·g^−1^; 11.43 µmol TE·g^−1^ and 38.95 µmol TE·g^−1^) and *L. angustifolius* (Lang4318: 3.11 mg GAE·g^−1^; 0.51 mg CE·g^−1^; 12.69 µmol TE·g^−1^ and 51.48 µmol TE·g^−1^). Notably, commercial varieties from Ecuador INIAP 450 Andino and INIAP 451 Guaranguito substantially surpassed *L. mutabilis* across all antioxidant parameters, establishing their potential as elite genetic resources. These phytochemicals are characterized by presenting a high antioxidant activity, meaning they neutralize free radicals in the human body, providing protective action against diseases related to oxidative stress [15,17,19,20].

According to Córdova-Ramos et al. [34] and Estivi et al. [19], the antioxidant activity in *L. mutabilis* determined by ABTS and FRAP assays is contingent upon extraction methodology, alkaloid content, and the genotypic identity of the cultivars. The antioxidant activity values observed in this study for the elite Ecuadorian accessions substantially exceed those reported by these authors in the two methods evaluated, FRAP (18.41 µmol TE·g^−1^) and ABTS (40.17 to 167 µmol TE·g^−1^), demonstrating the superior phytochemical potential of this germplasm collection. Genotypic variation emerged as the primary determinant of the observed variation in antioxidant capacity. Despite cultivation under uniform agro-environmental conditions, ABTS values exhibited substantial genotype-dependent variability, ranging from 212.47 µmol TE·g^−1^ (LmPron19) to 432.98 µmol TE·g^−1^ (INIAP 450 Andino), representing an approximately two-fold difference (Table 4).

### 2.5. Alkaloids

*Lupinus* species also contain antinutritional compounds, notably alkaloids, which necessitated careful germplasm selection based on food safety and palatability for human consumption. Genotypes exhibiting the lowest quinolizidine alkaloid concentrations were prioritized, as they fell within the “sweet” lupin category, requiring minimal debittering processing while mitigating the negligible risk of acute anticholinergic toxicity [17,31]. The alkaloid content across the evaluated *L. mutabilis* germplasm ranged from 0.04% to 1.77% (Table 5).

The lowest concentrations were detected in LmAnds16 and LmFR9s43 (0.04%), whereas INIAP 450 Andino (1.77%) and INIAP 451 Guaranguito (1.55%) exhibited the highest alkaloid contents. These findings align with the broader range reported by Carvajal-Larenas et al. for *L. mutabilis* (0.007–4.50%), though the genotypes evaluated herein did not exceed 2.00% alkaloid content, suggesting successful selection for lower alkaloid seeds. The considerable variation in alkaloid levels within *L. mutabilis* reflects both significant genetic variability and sensitivity to environmental and agronomic factors. Notably, low-alkaloid genotypes LmAnds16 and LmFR9s43 (0.04%) fall below the Ecuador consumption threshold of 0.07%, whereas the improved Ecuadorian commercial cultivars (INIAP 450 Andino and INIAP 451 Guaranguito) substantially exceed this limit and would require alkaloid elimination prior to human consumption [17]. By contrast, *L. albus* (0.06%) and *L. angustifolius* (0.10%) demonstrated considerably lower alkaloid contents, consistent with previously reported values by Carvajal-Larenas et al. [31].

Food regulatory standards vary markedly across regions: Australia and select European countries enforce a stringent threshold of 0.02%, whereas Ecuador permits up to 0.07%. Consequently, bitter lupin varieties require alkaloid detoxification before consumption, though conventional debittering processes typically achieve only partial alkaloid removal [19,25,31,34].

Debittering is a critical technological step that makes lupin consumption viable by reducing toxic alkaloids (lupanine: 35–55%; angustifolin: 15–25%; espartine: 10–20%; multiflorine, senatine, and others: 5–15%) to safe levels (<0.02% or 200 mg/kg, according to FAO/WHO regulations) for human and animal consumption [24]. The importance of this process lies in the neurological toxicity and cardiotoxic and neuromuscular effects resulting from consumption at high concentrations [17,31].

Technologically, debittering is carried out using different processes: aqueous soaking or leaching (90–140 h/4–6 cycles), steam cooking (60–90 h), and controlled fermentation (48–72 h), which allow for 95.0–99.9% elimination. The traditional aqueous method is the most widely used at the industrial level due to its high effectiveness (99.90%) in eliminating alkaloids. However, the application of the aqueous process generates unsustainable water consumption (300–500 L/kg) and causes significant losses of nutrients (protein: 8–12%; soluble and free amino acids: 40–60%) and phytonutrients (polyphenols, flavonoids, and isoflavones: 45–70%) [17,24,34].

Therefore, the development and selection of germplasm with low alkaloid content optimizes the post-harvest processing of lupin seeds, simultaneously preserving their nutritional and functional characteristics and reducing the environmental impact associated with conventional debittering methods.

### 2.6. Correlation Analysis

**Figure 1:**
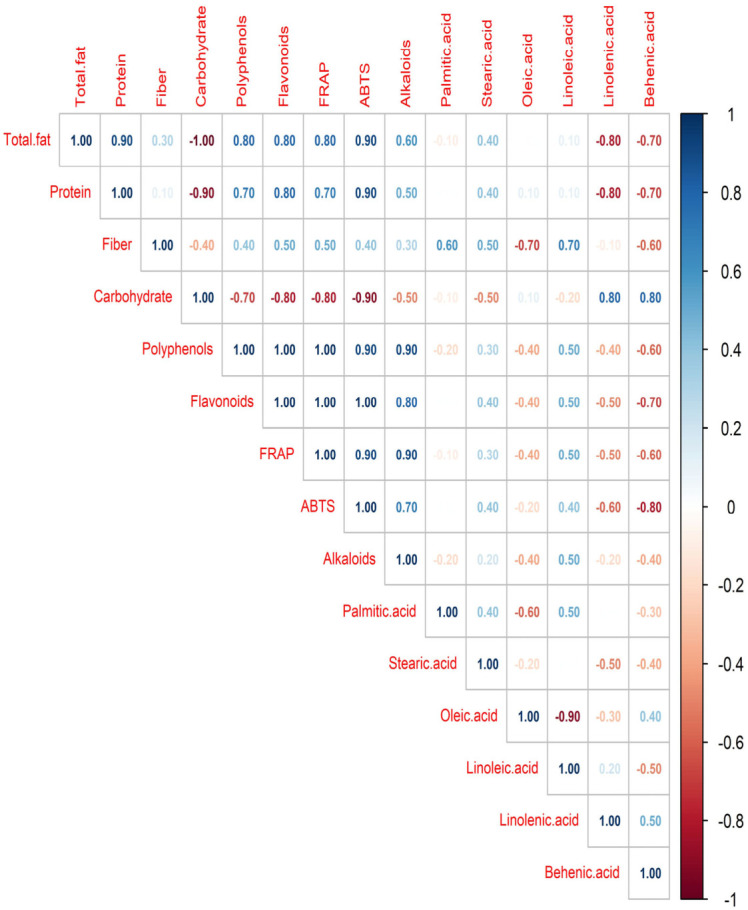
Pearson correlation matrix for the relationship between the studied variables of 12 Lupinus genotypes cultivated in Ecuadorian highlands.

The correlation matrix (Figure 1) revealed a complex pattern of associations among nutritional and phytochemical variables in *L. mutabilis* seed composition. Nutrient-level associations demonstrated strong positive correlations between fat and protein content (r = 0.90, *p* < 0.01), consistent with documented patterns of coordinated lipid and protein accumulation during seed filling. In marked contrast, carbohydrate content exhibited reciprocal negative associations with both fat and protein synthesis, constraining carbohydrate deposition.

Secondary metabolite patterns revealed that alkaloid content correlated positively with antioxidant capacity (ABTS and FRAP) and antioxidant components (flavonoids and polyphenols) with coefficients correlation ranging from r = 0.70 to r = 0.90 (*p* < 0.01). However, the alkaloid variable displayed strong negative correlations with protein and total fat (r = 0.70; *p* < 0.01). Low-alkaloid genotypes (LmAnds16 and LmFR9s43) exhibit reduced antioxidant capacity. Despite this trade-off, antioxidant levels in low-alkaloid genotypes remain competitive with those of other Lupinus cultivars reported in the literature [2,17,21,34,40]. The Pearson correlation matrix further revealed strong positive associations between total polyphenol content, total flavonoid content, and antioxidant activity measured by FRAP and ABTS (Figure 1). Total polyphenols were highly correlated with FRAP (r = 1.00, *p* < 0.01) and ABTS (r = 0.90, *p* < 0.01), while flavonoids exhibited equally strong correlations with FRAP (r = 1.00, *p* < 0.01) and ABTS (r = 1.00, *p* < 0.01). These results indicate that phenolic compounds are major contributors to the antioxidant capacity of the evaluated germplasm, consistent with previous studies in Lupinus species [19,34,37,41].

The resulting next generation of cultivars integrating maximal protein content, elevated phenolic compounds (FRAP and ABTS), concentrated micronutrients profiles (Zn, Fe, Ca) and minimized alkaloids (<0.1%) position *L. mutabilis* as a transformative crop capable of addressing global malnutrition while advancing sustainable agro-industrial development. Strategic deployment of superior genotypes represents a critical contribution to global nutrition security and health equity. These correlations validate the methodological coherence of antioxidant assays, as both FRAP and ABTS quantify the same phenolic reducing pool.

Notably, this finding corroborates prior evidence from comparative evaluations of major Lupinus species. Dalaram et al. [41], Estivi et al. [19] and Grela et al. [37] reported concordant results when evaluating Lupinus species (*L. albus*, *L. luteus*, *L. angustifolius*, and *L. mutabilis*) and multiple legumes: pea (*Pisum sativum*), common bean (*Phaseolus vulgaris*), and broad bean (*Vicia faba*). This consistency across diverse germplasm suggests that the flavonoid antioxidant relationship in lupine is a robust biochemical trait, likely reflecting coordinated transcriptional regulation of phenolic biosynthesis pathways [2,15,17].

A strong positive correlation (r = 0.90, *p* < 0.01) was identified between total protein and total fat content, consistent with values previously reported for *L. mutabilis* [41,42]. This robust association reflects coordinated genetic control during seed maturation, wherein enhanced allocation of photosynthetic products toward storage protein biosynthesis is inherently linked with concurrent triacylglyceride accumulation [2,15,35].

Coefficients of 0.40 and 0.50 are significant at *p* < 0.05; coefficients of 0.60 are significant at *p* < 0.01 and coefficients between 0.70 and 0.90 are significant at *p* < 0.01; blank cells represent non-significant coefficients.

This coordinated metabolic allocation represents an adaptative strategy optimizing nutrient density, a characteristic of particular significance for cultivars targeted toward food and feed applications [6]. Conversely, protein content demonstrated a strong reciprocal negative correlation with carbohydrates (r = −0.90, *p* < 0.01), supporting prior observations by Córdova-Ramos et al. [34] and Dalarman et al. [41]. This antagonism reflects a metabolic dilution effect of genotypes allocating greater proportions of dry mass to protein synthesis, necessarily reducing structural carbohydrate deposition. This relationship has critical implications for developing lupine varieties targeting specific nutritional niches: formulations prioritizing elevated protein with low glycemic index require simultaneous selection for both elevated protein and reduced carbohydrate content, a constraint that warrants explicit consideration in breeding objectives [17,34].

The alkaloid concentration exhibited a moderate positive correlation with total protein content (r = 0.50, *p* < 0.01), consistent with findings previously documented by Rodríguez-Ortega et al., [14]. This association indicates genetic linkage or coordinated metabolic regulation between protein synthesis and alkaloid biosynthesis pathways during seed development. However, substantial genotypic variation exists regarding this relationship, providing opportunities for selective breeding. Notably, the accessions LmAnds16 (44.89% protein, 0.04% alkaloids) and LmFR9s43 (42.13% protein, 0.04% alkaloids) demonstrate that the decoupling of alkaloid accumulation from high protein content is achievable [26,42]. These accessions represent exceptional germplasm for the Ecuadorian Crop Improvement Program, demonstrating the feasibility of developing low-alkaloid varieties while maintaining enhanced nutritional protein concentration. Critically, even fully domesticated, low-alkaloid (debittered) *Lupinus* genotypes retain substantial compositional advantages relative to competing legume species, preserving their appeal as alternative protein sources [17]. This finding reinforces the strategic value of targeted germplasm selection in developing lupine cultivars optimized for both palatability and nutritional quality.

Fatty acid composition exhibited distinct partitioning patterns. Linolenic acid showed strong negative associations with protein, fat, and ABTS content (r = −0.60 to −0.80, *p* < 0.01–0.05), but conversely demonstrated strong positive correlation with carbohydrates (r = 0.80, *p* < 0.01). Linoleic and oleic acids exhibited competitive antagonism (r = −0.90, *p* < 0.01), reflecting documented desaturase pathway constraints; moreover, oleic acid correlated positively with fiber (r = −0.70, *p* < 0.01), while linoleic acid correlated positively with fiber (r = 0.70, *p* < 0.01). Linoleic and oleic acids exhibited competitive antagonism (r = −0.90, *p* < 0.01), reflecting documented desaturase pathway constraints; moreover, oleic acid showed strong negative correlation with fiber content (r = −0.70, *p* < 0.01), while linoleic acid correlated positively with fiber (r = 0.70, *p* < 0.01).

In contrast, saturated fatty acids (palmitic and stearic) exhibited no significant associations with any evaluated variable, suggesting independent genetic control over their biosynthesis. Behenic acid similarly displayed negative correlations with protein, fiber, flavonoids, and ABTS (*p* < 0.01). Behenic acid (C22:0, saturated long chain) similarly exhibited strong negative correlations with protein (*p* < 0.01), fiber (*p* < 0.01), flavonoids (*p* < 0.01), and ABTS (*p* < 0.01), suggesting that high behenic genotypes occupy a distinct metabolic space characterized by lower structural integrity and reduced secondary metabolism investment. This competitive relationship has direct implications for lipid quality and oxidative stability: genotypes enriched in oleic acid (monounsaturated, C18:1n9C) would exhibit superior shelf-life stability and oxidative resistance, while those elevated in linoleic acid (polyunsaturated, C18:2) would offer enhanced nutritional profiles for omega-3 and omega-6 balance in human nutrition. This finding enables targeted selection for lipid-based product specifications of oleic-enriched varieties for culinary applications and linoleic-enriched selections for functional food development [15,28,36].

### 2.7. Multivariate Analysis

#### 2.7.1. Principal Component Analysis (PCA)

**Figure 2:**
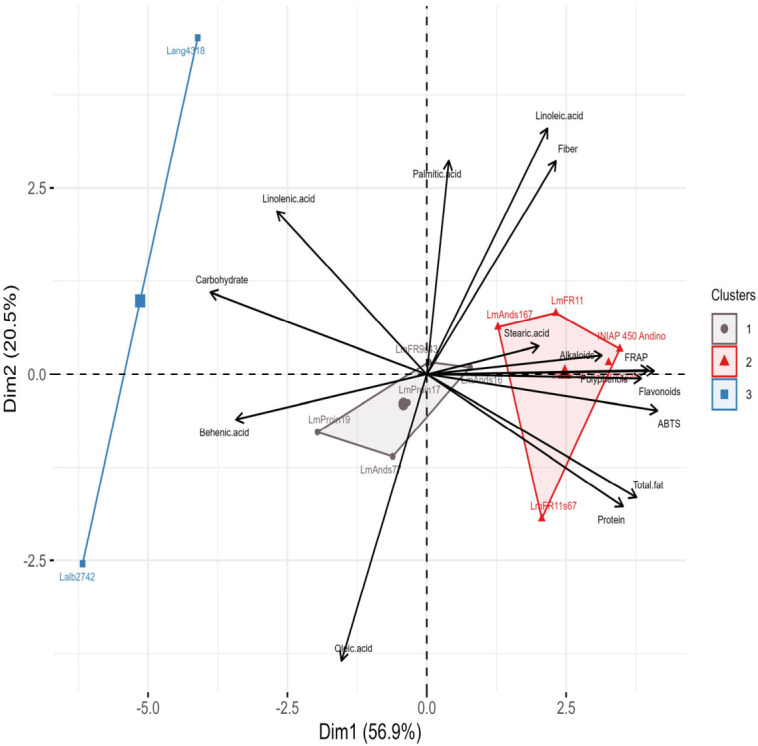
Multivariate phytochemical stratification of Lupinus germplasm: principal component analysis (PCA) for phenotypic clustering, secondary metabolite, and lipid compositional variance across domesticated species and Andean-origin breeding lines: *L. mutabilis*, *L. albus* and *L. angustifolius*.

Principal component analysis revealed that the first two components explain 77.4% of the total phenotypic variation observed across the evaluated germplasm collection (Figure 2). This dimensional reduction proves statistically sufficient to capture the underlying patterns of phytochemical and nutritional co-variability, thereby validating the biological relevance of the principal component’s structure.

The substantial explanatory PC1 (56.9%) and PC2 (20.5%) indicate that the measured biochemical traits exhibit well-defined clustering patterns intrinsic to the genetic architecture of the evaluated Lupinus genotypes [2]. The loading structure of PC1 reveals a fundamental biochemical trade-off axis. Alkaloids contents, flavonoids, FRAP antioxidant activity and carbohydrate accumulation are positively correlated with PC1, whereas polyphenols, ABTS antioxidant capacity, total fat, protein and behenic acid exhibit negative correlations. Notably, PCA demonstrates an inverse relationship between protein and total fat contents relative to carbohydrate accumulation, a finding consistent with fundamental partitioning of photo-assimilates toward storage compounds versus structural constituents [15].

The positive relationship observed between protein total fat and antioxidant capacity components (Figure 2) suggests coordinated biosynthetic regulation of these nutritionally favorable traits. More significantly, the evident association between alkaloid content and antioxidant capacity, corroborated by this correlation matrix, indicates that secondary metabolite production may be functionally linked to the oxidative stress response mechanisms in these Andean legume accessions [17].

The PC2 segregates genotypes based on fatty acid chain-length diversity. Oleic acid content demonstrated negative correlation, while linolenic acid, palmitic acid and fiber content are positive correlations. This configuration reveals a critical inverse relationship between polyunsaturate acids (linolenic acid and linolenic) and oleic acid and insoluble fiber content, suggesting distinct metabolic strategies governing lipid deposition and cell wall biogenesis across the germplasm [2,15]. Behenic acid (C22:0) exhibits strong negative correlation with stearic acid, alkaloid content, protein and antioxidant capacity components, a pattern indicating potential metabolic competition between saturated fatty acids, elongation pathways and secondary metabolite synthesis.

Conversely, stearic acid demonstrates positive associations with alkaloid contents and antioxidant activity components, echoing the patterns observed in bivariate correlation analysis (Figure 2). PCA ordination unequivocally demonstrates that *L. mutabilis* materially diverges from *L. albus* (Lalb2742) and *L. angustifolius* (Lang4318) in their multivariate biochemical profiles. This species-level differentiation corroborates previous findings reported by Estivi et al. [19], who documented analogous segregation patterns in antioxidant activity across the four cultivated species of *Lupinus.* Furthermore, Czubinski et al. [43] reported congruent results when applying PCA to the combination of antioxidant capacity, fatty acid and protein content across these four *Lupinus* species, thereby providing independent validation of the robust taxonomic signal inherent in these biochemical traits.

These native materials cluster within the positive region (PC1), associating predominantly with elevated linoleic and linolenic acids (PUFA) levels and balanced protein profiles. This positioning reflects the nutritional quality attributes of Andean sweet lupine germplasm and substantiates their potential as sources of beneficial allelic variation for crop improvement programs.

The LmAnds accessions demonstrate variable distribution across the central ordination space, particularly LmAnds167, displaying elevated secondary metabolite accumulation alkaloids and polyphenols. This pattern suggests underlying genetic heterogeneity with respect to embolic regulation and secondary metabolism investment within the *L. mutabilis* subspecies complex. Likewise, these genotypes LmFR (LmFR11, LmFR11s67 and LmFR9s43) and LmProim (LmAnds16, LmAnds77 and LmAnds167) segregate toward positive PC1 values with variable protein content expression, indicative of significant genetic differentiation in proteomic efficiency and nitrogen metabolic partitioning across the evaluated germplasm.

The multivariate analysis reveals that genotype-specific biochemical signatures reflect distinct metabolic strategies rather than random phenotypic variation. The segregation along PC2 represents an important axis of agronomic variation, as it directly impacts both nutritional quality (fatty acids and fiber content) and feed value in a legume crop context. The apparent antagonism between oleic acid and polyunsaturated fatty acid accumulation may reflect differential regulation of desaturase enzyme families, a mechanistic insight warranting further investigation through comparative transcriptomic studies.

Critically, the positive association between alkaloid content and antioxidant activity, while potentially advantageous for nutraceutical applications, necessitates careful evaluation of antinutritional compound levels in breeding programs targeting human consumption.

INIAP 450 Andino emerges as an exemplary genotype, combining favorable nutritional attributes with elevated PUFA, balanced protein and moderate alkaloid levels, positioning it as an optimal candidate for advanced breeding initiatives focused on sustainable crop intensification in Andean agroecological systems.

#### 2.7.2. Cluster Analysis

**Figure 3:**
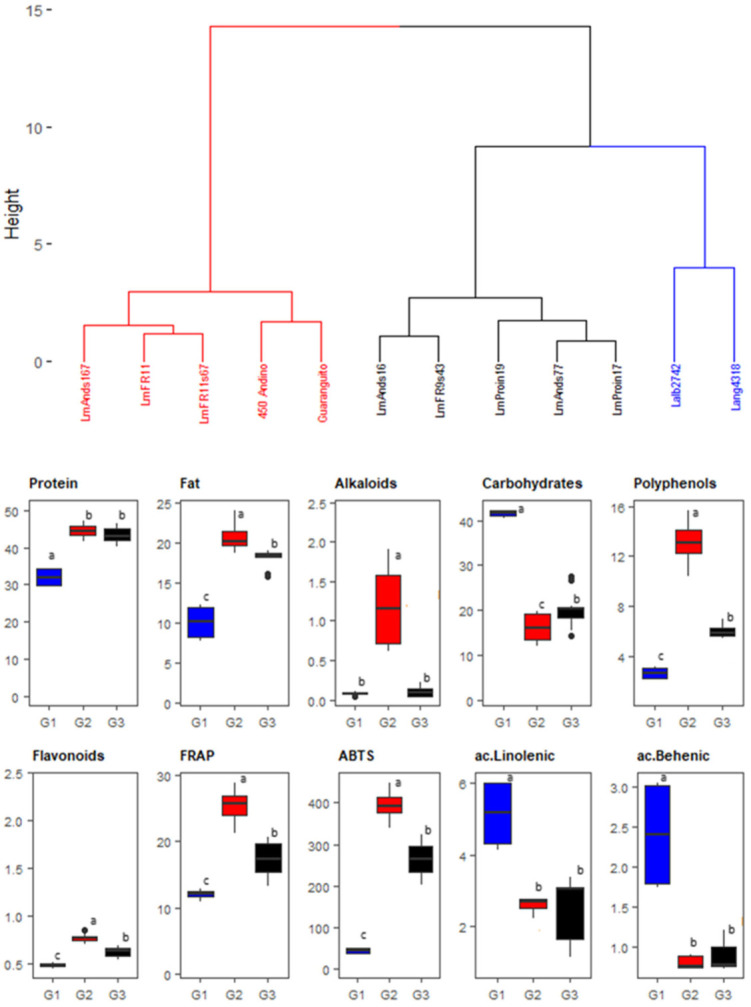
Identification of three phenotypic clusters (G1, G2, G3) in Lupinus germplasm: dendrogram-based stratification and biochemical phenotyping revealing species differentiation and intraspecific metabolic diversity of seeds from 12 cultivars planted in the Ecuadorian highlands. a, b, c: Significant differences (*p* < 0.05) using Tukey’s test at 0.05.

Agglomerative hierarchical clustering combined with the Elbow method determined three phenotypically and genetically distinct clusters within the evaluated Lupinus germplasm (Figure 3).

This tripartite clustering architecture substantiates and extends the multivariate patterns observed in PCA ordination, providing a complementary classification framework grounded in Euclidean distance metrics. The dendrogram structure reveals clear hierarchical differentiation, with the primary bifurcation separating the control cultivars *L. albus* and *L. angustifolius* from the sweet Lupinus from INIAP germplasm complex at a substantial phylogenetic distance.

The first group (cluster 1: introduced germplasm control), comprising *L. albus* (Lalb2742) and *L. angustifolius* (Lang4318) cultivar, exhibited a taxonomically distinct position characterized by a fundamentally different biochemical phenotype relative to the *L. mutabilis* germplasm. These genotypes exhibit significantly lower levels of protein (<35%), total fat (<15%), alkaloids and antioxidant activity (FRAP and ABTS), flavonoids, polyphenols and higher carbohydrate content. This phenotypic profile reflects the differential domestication history and agronomic selection pressures applied in control cultivar Lupinus, which have been historically cultivated for high carbohydrate yield rather than nutraceutical quality attributes.

The suppressed antioxidant activity and markedly reduced alkaloid accumulation in this cluster indicate fundamentally divergent metabolic trajectories governing secondary metabolism. Notably, the insignificant variation of fiber content, saturated (palmitic acid and stearic acid) and unsaturated fatty acids (oleic and linolenic acid) across the three clusters suggest that the primary fatty acid composition and raw fiber content represent conserved traits at the species level, with secondary modifications driven by genotype-specific regulatory mechanisms.

The second group (cluster 2: high value nutraceutical) comprises the commercial Ecuadorian cultivars INIAP 450 Andino, and INIAP 451 Guaranguito, the promising pink flowers line (LmFR11) and the selected Andean accessions LmAnds167 and LmFR11s67. This assemblage represents the elite phenotype for simultaneous optimization of multiple nutritional and phytochemical quality attributes. Cluster 2 genotypes are distinguished by their substantially elevated protein content (>40%), markedly reduced carbohydrate allocation (>30%) and exceptionally high total fat content (>18%), positioning them optimally for both human nutritional supplementation and animal feed applications [15].

Critically, genotypes of cluster 2 are characterized by pronounced elevation in antioxidant activity values, substantially exceeding those of clusters 1 and 3. This superior antioxidant by FRAP and ABTS appears functionally integrated with the markedly elevated alkaloid content (0.50 to 2.00%), consistent with the biochemical association observed in the matrix correlation and PCA analyses. The elevated polyphenol levels further substantiate the enhanced secondary metabolite accumulation in this cluster, suggesting coordinated upregulation of phenylpropanoid and terpenoid biosynthetic pathways [2,19,41].

The co-expression of these favorable traits is coupled with the commercial validation of the INIAP varieties of positions in cluster 2 (Figure 3) as the primary target genotype pool for advanced breeding initiatives focused on crop intensification and value chain development. The inclusion of both commercial cultivars and elite breeding lines indicates that substantial genetic gains can be achieved through continued selection within this cluster.

The third group (cluster 3: low alkaloid) consists of an *L. mutabilis* line with intermediate nutritional quality, which encompasses the introduced genotypes LmProin17 and LmProin19, together with select Andean lines LmAnds16, LmAnds77 and LmFR9s43. These genotypes maintain the high protein content characteristic of sweet *L. mutabilis* (>40%) and reduced carbohydrate allocation (>30%) with intermediate levels along the secondary metabolite accumulation gradient (antioxidant capacity, flavonoids, and polyphenols) and macronutrient (fat and carbohydrates).

Most significantly, cluster 3 is distinguished by exceptionally low alkaloid contents (0.04 to 0.23%), representing a reduction of approximately 90% relative to cluster 2.

The functional properties (antioxidant capacity FRAP and ABTS, total polyphenol) of cluster 3 exceed those of cluster 1 but remain substantially lower than cluster 2, indicating partial suppression of antioxidant biosynthesis concurrent with alkaloid reduction. Total fat content in cluster 3 (15–18%) is intermediate between cluster 1 and cluster 2, suggestions distinct metabolic regulation governing lipid deposition. Flavonoid content similarly reflects this intermediate phenotype.

The pronounced alkaloid reduction in cluster 3, while potentially advantageous for human food applications requiring minimal antinutritional compound content, represents a substantial forfeit of the antioxidant and nutraceutical attributes associated with elevated secondary metabolism [22,42]. This phenotypes stratification suggests that alkaloids biosynthesis acts as a coordinating hub regulating broader secondary metabolite accumulation, warranting future investigation into the transcriptomic and enzymatic determinants of this metabolic coordination [26].

The absence of statistically significant variation in fiber content palmitic acid, stearic acid, oleic acid and linolenic acid across the three clusters indicates that these variables represent conserved traits insufficiently polymorphic for germplasm discrimination. This pattern suggests strong historical selection or constraints on the allelic diversity governing these biochemical pathways across the cultivated Lupinus germplasm. Conversely, the robust differentiation among clusters for protein, fat, alkaloids, carbohydrates and antioxidant capacity components identifies these as primary drivers of phenotypic diversity and appropriate selection criteria for breeding programs.

Multivariate analysis of the evaluated Lupinus germplasm reveals a complex phenotypic architecture that stratifies genetic diversity into three functional groups, each with differential attributes for different value chains and agrifood applications. The genotypes were grown under uniform agronomic management and met the global challenge of providing dietary solutions linked to the diet–health–environment trilemma [32,44,45].

Their nutritional contribution, with a high percentage of protein (>40%), carbohydrates (<30%), fats (15–18%), and fiber, make them a potential food to incorporate into the diet, demonstrating an intrinsic genetic predisposition toward the simultaneous synthesis of proteins, bioactive secondary metabolites, and antioxidant compounds [2,24]. The content of antioxidant components such as polyphenols, flavonoids, and fatty acids (PUFA and MUFA) promotes the proper functioning of the body and strengthens the immune system thanks to their bioactive properties [2,24,34,45]. From an environmental point of view, all Lupinus genotypes contribute through biological nitrogen fixation, reduced dependence on synthetic fertilizers, improved soil properties, and a comparatively lower water footprint compared to conventional protein legumes [2,14,17].

These characteristics highlight the remarkable nutritional and phytochemical value of L. mutabilis and support its potential use as a nutrient-dense ingredient in functional food formulations. Furthermore, its favorable nutritional profile makes it a valuable genetic resource for breeding programs aimed at improving the nutritional quality and added value of lupin-derived food products [16,38,44].

## 3. Materials and Methods

### 3.1. Plant Material

**Figure 4:**
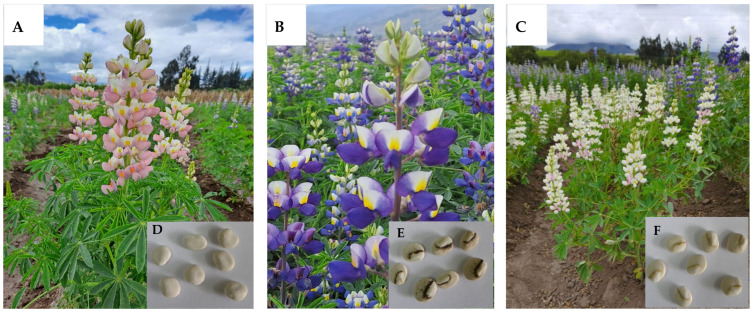
Grain and flower color variation among the evaluated *Lupinus mutabilis* genotypes. Source: Rodriguez et al., [14] and Rivera et al., [11]. (**A**) White grain: LmAnd s77, LmFR11, LmFR11 s67, INIAP 450 Andino, INIAP 451 Guaranguito; (**B**) white grain with black eyebrow: LmAnd s16, LmFR9 s43, LmProin 17; (**C**) white grain with brown eyebrow: LmAnd s167, LmProin 2019; (**D**) pink flowers: LmFR11, LmFR9 s43; (**E**) bluish flowers: LmAnd s16, LmAnd s77, LmAnd s167, INIAP 450 Andino, INIAP 451 Guaranguito, LmProin 17, LmProin 2019; (**F**) flowers initially white, turning pink at later developmental stages: LmFR11 s67.

From a total of 200 accessions (Figure 4) in the active germplasm bank (stored at 5 °C) of the Improvement Program of the National Institute of Agricultural Research of the Santa Catalina Experimental Station (INIAP-EESC), 10 genotypes were selected based on plant architecture (mechanization), crop cycle (150 to 210 days to harvest) and alkaloid content (0.04% to 2%) [11,16].

The high quinolizidine alkaloid (QA) content represents a limiting factor for the utilization of *Lupinus mutabilis* Sweet in the food industry, as it imparts a bitter taste and may exhibit acute anticholinergic toxicity, thereby necessitating breeding improvements through hybridization to develop novel low-alkaloid [17] grain varieties at INIAP Santa Catalina Experimental Station (E.E. S. C.) from INIAP. In addition, 2 introduced genotypes from Bolivia were included, *L. albus* (Lalb2742) and *L. angustifolius* (Lang4318), resulting in the analysis of a total of 12 *Lupinus* genotypes, comprising advanced breeding germplasm, improved varieties and introduced genotypes (Table 6).

### 3.2. Experimental Site

The Lupinus germplasm samples analyzed were planted at the Santa Catalina Experimental Station (INIAP-EESC) to maintain consistent environmental conditions, agronomic management and climatic conditions. Table 7 shows the location of the experiment site and the soil conditions for planting [14]. In addition, the results of the soil analysis (pH, macro- and microelements), measured eight days before planting, are shown. The soil analysis was carried out at the Soil, Plant and Water Analysis Laboratory of INIAP-EESC. Due to the soil quality, planting was carried out directly, without fertilization.

### 3.3. Sample Preparation

The pods of each genotype were manually harvested from the experimental plots, then manually threshed. The seeds were then placed in paper bags with their respective identification and transported to the Nutrition and Quality Laboratory (ISO/IEC 17025 certification [46]) at INIAP-EESC. For laboratory analysis, a sample of 100 g of dry seeds was taken and subjected to a grinding process using a Retsch ZM 200 ultracentrifugal mill (Hann, Germany) until a particle size of <1 mm was obtained. The ground samples were stored in an airtight container in a cool, dry place prior to analysis.

### 3.4. Chemical Reagents

Deionized water was obtained through a MILLI-Q Academic water purification system (Millipore, Sao Paulo, Brazil). The Standards for (+) catechin, gallic acid, ABTS (2,2-azinobis-3-ethyl-benzothiazoline-6-sulfonic acid), and Trolox (6-hydroxy-2,5,7,8-tetramethylchroman-2-carboxylic acid) were obtained from Sigma Aldrich (St. Louis, MO, USA). The Fame Mix C4 to C24 standard was obtained from Supelco (PA, USA) and the standards for macroelements (Ca, Mg, Na, K and P), microelements (Cu, Fe, Mn and Zn), and analytical grade solvent reagents were obtained from Merck (Darmstadt, Germany).

### 3.5. Proximal Composition

#### 3.5.1. Protein

The protein content was determined using the AOAC 2001.11 methodology. For this, 1 g of sample was weighed in a 250 mL digestion tube; 2 Copper catalyst tablets (3.5 g K_2_SO_4_ and 0.4 g CuSO_4_·5H_2_O) and 15 mL of concentrated sulfuric acid were added. The tubes were immediately placed in a digestion block and heated at 400 °C for 1 h. After this, the tubes were subsequently cooled for 1 h and placed in an automatic protein analyzer (FOSS Kjeltec model 8400; Hillerod, Denmark) where distillation and titration were performed. The results were expressed as grams of protein per 100 g of dry sample [40].

#### 3.5.2. Fat

The fat content was evaluated through continuous extraction with organic solvent using the Soxhlet method, as proposed by Flor-Unda et al. [40]. For this, 5 g of sample was weighed into a cellulose thimble (33 × 88 mm), covered with cotton, and subjected to an extraction process with 200 mL of hexane at 90 °C in a Soxhlet extraction system (Selecta, Barcelona, Spain) for 16 h. The thimbles with the defatted sample were removed from the extraction system and the solvent was recovered. The distillation flasks containing the lipid fraction were placed in an oven (Precision Scientific, Chicago, IL, USA) at 105 °C for 2 h to remove solvent residue. Then, they were cooled in a desiccator for 2 h and weighed using a Shimadzu AUX 220 analytical balance (Kyoto, Japan). The results were reported as grams of fat per 100 g of dry sample.

#### 3.5.3. Raw Fiber

To determine the total fiber, the AOAC 978.10 method was followed. For this, 1 g of sample was weighed in porous glass crucibles (100 µm) and placed in the FOSS Fibertec 8000 equipment (Hillerod, Denmark). Once the heater reached 120 °C, the sample was subdued to acid digestion (1.25% *v*/*v* sulfuric acid solution) and alkaline digestion (1.25% *w*/*v* NaOH solution) for 1 h each. After this time, the samples were washed with distilled water. The crucibles with the digested samples were then removed from the equipment and placed in a Lab-Line Imperial V convection oven (USA, IL) at 105 °C for 1 h, followed by a calcination process at 500 °C for 8 h.

Finally, the samples were placed in a desiccator, cooled, and the weights of the dry and the calcined samples were recorded. The results were expressed as grams of fiber per 100 g of dry sample [40].

#### 3.5.4. Ash

Ash content was determined using AOAC method 923.03. For this, 1 g of sample was weighed into 25 mL capacity porcelain crucibles and then underwent a calcination process at 500 °C in a Thermolyne 48000 muffle (Dubuque, IA, USA) for 12 h. The calcined samples were cooled for 1 h and transferred to a desiccator. The weight of each crucible was recorded, and the ash content was calculated by weight difference. The results were expressed in grams of ash per 100 g of dry sample [40].

#### 3.5.5. Moisture

The moisture content was determined using AOAC method 925.40. For this, 0.5 g of sample was weighed in aluminum capsules and immediately subjected to a drying process in a Lab-Line Imperial V convection oven (USA, IL) at a temperature of 105 °C for 16 h. Subsequently, the capsules were transferred to a desiccator and cooled for 2 h. Finally, each capsule with the dry sample was weighed and the moisture content was determined by weight difference. The results were expressed as a percentage of moisture in each sample [40].

### 3.6. Fatty Acid Profile

#### 3.6.1. Sample Preparation

The fatty acid profile was performed using the methodology proposed by Samaniego et al. [47]. For this, 0.05 g of chocho fat was weighed in a screw-cap test tube, 1 mL of 0.5 M KOH/methanol solution was added. The mixture was heated using a water bath at 90 ° C for 10 min. Subsequently, 4 mL of HCl/methanol (4:1 *v*/*v*) was added and the sample was heated again using a water bath at 90 ° C for 25 min. The sample was immediately cooled to room temperature, followed by the addition of 2 mL of double-distilled water and 3 mL of hexane. The mixture was shaken for 30 s, and the organic phase was collected. This process was repeated twice more to recover all the fatty acid methyl esters. The organic extract was dried under a nitrogen stream at 25 °C and reconstituted with 2 mL of HPLC grade hexane.

#### 3.6.2. Quantification

The fatty acid analysis was conducted using an Agilent Technologies 7890A gas chromatograph with flame ionization detector (FID), controlled by the ChemStation Productivity software (MSD ChemStation Productivity E.02.00.493; Agilent Technologies, Waldbronn, Germany). Separation was performed on a SPTM 2560 fused silica capillary column (100 m × 0.25 mm I.D., 0.2 µm film thickness). For the analysis, 2 µL of the sample was injected into Split mode at 260 °C. The GC was programmed with helium as the carrier gas at a flow rate of 1.07 mL/minute. The column oven was programmed with the following conditions: 140 °C (5 min) to 240 °C (5 min) with a ramp rate of 4 °C/min. Identification was performed by comparing peak retention times with their respective standards, using a FAME Mix standard of C4 to C24 as a reference. Quantification was carried out using the external standard method, comparing the sample area with the area of its respective standard. The results were expressed as grams of each fatty acid per 100 g of fat [47].

### 3.7. Minerals

#### 3.7.1. Sample Preparation

In a muffle (Thermolyne 48000; Dubuque, IA, USA), 1 g of sample was submitted to a calcination process at 400 °C for 12 h. It was then cooled in a desiccator and placed on a heating plate (Witeg; Wertheim, Germany), to which 5 mL of HCl (37%) and 10 mL of Type I water (18.2 MW cm) were added. The sample was digested at 100 °C until its volume was reduced by half. The sample was filtered in a 100 mL volumetric flask using Whatman quantitative ash-free filter paper (Maidstone, UK) and brought to a final volume of 100 mL with Type I water [48].

#### 3.7.2. Quantification of Macroelements

For the determination of macroelements such as Ca and Mg, the methodology used by Viera et al. (2022) was followed by adding 0.5 mL of 1% lanthanum solution to 4.5 mL of the sample [48]. For Na and K, 0.5 mL of 1% lithium solution was added to the same volume of sample. The absorbance was measured using an Atomic Absorption spectrophotometer (Shimadzu AA 7000; Tokyo, Japan). The analysis of P was performed by taking 0.5 mL of sample, adding 4 mL of Type I water, and then 0.5 mL of 1% ammonium molybdovanadate solution. The absorbance was measured using a UV–visible spectrophotometer (Shimadzu UV 2600; Tokyo, Japan).

#### 3.7.3. Quantification of Microelements

For the determination of microelements (Cu, Fe, Mn, and Zn), the absorbance was measured on 5 mL of the sample using an Atomic Absorption spectrophotometer (Shimadzu AA 7000; Tokyo, Japan). Quantification was performed by comparing sample absorbances against calibration curves constructed for each element. Results were expressed as mg of each element per kilogram (mg∙kg^−1^) of dry weight (DW) [48].

### 3.8. Functional Properties

#### 3.8.1. Extraction of Bioactive Compounds

The extraction of antioxidants was done by weighing 0.3 g of dry sample into 15 mL polyethylene centrifuge tubes, then adding 5 mL of the extraction solution (methanol/water/formic acid 70/30/0.1 *v*/*v*/*v*). The mixture was homogenized by shaking in a FastPrep-24 device (Irvin, CA, USA) for 1 min and 10 min in a Cole-Parmer 8892 ultrasonic bath (Chicago, IL, USA). The sample was then centrifuged for 10 min at 5000 rpm and 5 ° C in a Sigma 4–16KS centrifuge (Osterode am Harz, Germany). The supernatant (extract) was transferred to a 25 mL amber volumetric flask. This process was repeated four times to ensure total extraction of the antioxidant compounds of the hydrophilic fraction [48].

#### 3.8.2. Total Antioxidants

##### Total Polyphenols

Total polyphenols were quantified using the methodology described by Samaniego et al. [47]. A 1 mL aliquot of diluted extract was placed in a test tube, to which 6 mL of distilled water and 1 mL of Folin & Ciocalteu’s reagent was added, and the mixture was allowed to rest for 3 min. Then, 2 mL of 20% Sodium carbonate was added, and the sample was heated to 40 °C for 2 min. This reaction formed a blue chromophore, which was analyzed in a Shimadzu 2600 UV/VIS spectrophotometer (Japan, Tokyo) at a wavelength of 760 nm. The results were expressed as milligrams of gallic acid equivalents per gram of dry sample (mg GAE·g^−1^ DW).

##### Total Flavonoids

The total flavonoid content was evaluated using the method developed by Llerena et al. [49]. A 1 mL aliquot of the diluted extract was placed in a test tube, to which 4 mL of distilled water was added, and the mixture was homogenized. Then, 0.3 mL of 5% Sodium nitrite was added, and after 5 min, 0.3 mL of 10% aluminum chloride was added, followed by another 5 min of resting. Consecutively, 2 mL of 1 N NaOH was added, obtaining a pink chromophore. Finally, the solution was brought to a final volume of 10 mL with distilled water. This solution was analyzed in a UV/VIS spectrophotometer (Shimadzu 2600; Tokyo, Japan) at a wavelength of 490 nm. The results were expressed as milligrams of catechin equivalents per gram of dry sample (mg CE·g^−1^ DW).

#### 3.8.3. Antioxidant Activity

##### ABTS^+^ Method

The antioxidant activity was evaluated using the ABTS method, following the methodology described by Viera et al. [48]. A solution of ABTS^+^ (7 mM) was mixed with a Potassium persulfate (2.45 mM) solution in a 1:1 ratio in an amber flask and allowed to rest for 16 h. The following day, the absorbance of the prepared ABTS^+^ working solution was measured and it was diluted with phosphate buffer to achieve an absorbance of 1.1 ± 0.01 at 734 nm. Then, 200 µL of the diluted extract was placed in a test tube, to which 3.8 mL of the ABTS^+^ working solution was added. The mixture was left to rest for 45 min, and the absorbance of the resulting reactions was determined using a spectrophotometer (UV/VIS Shimadzu 2600; Japan, Tokyo) at a wavelength of 734 nm. The results were expressed as µmol Trolox equivalent per gram of dry sample (µmol TE·g^−1^ DW).

##### FRAP Method

The Ferric Reducing Antioxidant Power (FRAP) was determined using the method described by Llerena et al. [49]. A 1 mL of sample was placed in a test tube and 2.5 mL of pH 6.6 buffer and 2.5 mL of a 1% Potassium ferrocyanide solution was added. The mixture was shaken and immediately incubated in a water bath at 50 °C for 20 min. Then, 2.5 mL of 10% trichloroacetic acid, 2.5 mL of water and 0.5 mL of 1% FeCl_3_ were added. Finally, it was homogenized in a vortex and after 30 min of resting in the dark, a green ferrous chloride–Potassium ferrocyanide complex was formed. This complex was analyzed using a Shimadzu 2600 UV/VIS spectrophotometer (Tokyo, Japan) at 700 nm. The results were expressed as µmol Trolox equivalent per gram of dry sample (µmol TE·g^−1^ DW).

### 3.9. Alkaloids

#### 3.9.1. Extraction

The extraction of alkaloids from *Lupinus* was carried out by placing 0.2 g of defatted sample into 15 mL polyethylene centrifuge tubes. To this, 5 mL of 5% trichloroacetic acid was added. The mixture was homogenized for 1 min by shaking in a FastPrep-24 equipment (Irvin, CA, USA) and placed for 10 min in a Cole-Parmer 8892 ultrasonic bath (Chicago, IL, USA). Afterwards, the mixture was centrifuged at 5000 rpm for 10 min at 5 °C in a Sigma 4–16KS centrifuge (Osterode am Harz, Germany). The supernatant was carefully separated in another centrifuge tube, and this procedure was repeated two more times to ensure complete extraction of quinolizidine alkaloids. In each extraction cycle, 0.5 mL of the supernatant was transferred to a test tube, and four drops of Dragendorff’s reagent were added to confirm the presence of alkaloids, indicated by the formation of brown precipitate (positive reaction) [50].

#### 3.9.2. Quantification

For the quantification of alkaloids, the lupin extract was transferred into a separatory funnel, neutralized with 1 mL of 10 M NaOH, and 15 mL of dichloromethane was added. The organic phase was collected in a 50 mL centrifuge tube and evaporated to dryness in a water bath at 50 °C under a stream of nitrogen. The extract was then re-dissolved in 5 mL of 0.01 N sulfuric acid, and two drops of methyl red indicator were added. The excess acid was titrated with a 0.01 N NaOH solution. The results were expressed as grams of lupinin equivalent per 100 g of dry sample [39].

### 3.10. Statistical Analysis

The data obtained from the duplicate analytical determinations were analyzed using descriptive statistics (arithmetic mean and standard deviation) and multivariate analysis (Pearson correlation and PCA). For univariate analysis, a one-way analysis of variance (ANOVA) was performed at a 95% confidence interval, and a Tukey test at 95% was used to determine differences between means. Through multivariate statistics, data analysis was performed using R software version 4.4.0 (R Core Team, 2024). Pearson correlation coefficients were calculated with a significance level of α = 0.05 to analyze the relationship between two variables. Principal component analysis (PCA) and the clustering analysis were done using the K-means method to analyze the data and compare their results [35]. PCA was used to visualize the relationship between variables and their association with *Lupinus* germplasm, while the clustering analysis grouped similar individuals, with the number of clusters defined using the Elbow method [51]. Consequently, the information obtained from these two methods was complementary.

## 4. Conclusions

Andean *Lupinus mutabilis* seeds exhibit a nutritionally favorable composition characterized by a protein content exceeding 40%, raw fiber, potential antioxidant capacity (polyphenols and flavonoids) and micronutrients (Zinc, Iron, Calcium, Potassium). The germplasm also contains bioactive alkaloid of pharmacological potential interest, together with a fatty acid profile favoring unsaturated forms, particularly oleic acid.

The lupine genotype collection, stratified into three phenotypically distinct commercial categories, shows a broad range of biochemical and nutritional contents. INIAP 450 Andino and INIAP 451 Guaranguito alongside high-performing breeding lines (LmFR11, LmAnds167 and LmFR11s67) comprises elite Ecuadorian varieties, characterized by highest antioxidant activity in vitro, for development of pharmaceutical products and nutraceutical foods.

LmAnds16, LmAnds77, LmFR9s43, LmProin17, and LmProin19, whilst maintaining robust protein content, represent seeds safety, characterized by pronounced alkaloid suppression (90%), optimally deployed for direct human consumption and vulnerable populations (0.04–0.23%). This allows the identification of the best genotypes for the development of new fortified varieties for direct consumption or for agro-industrial use. *L. albus* (Lalb2742) and *L. angustifolius* (Lang4318) represent substantial protein content, maximized polyphenol and flavonoid concentrations, and unique alkaloid biosynthesis with potential pharmacological applications.

Targeted selection within the INIAP breeding program has reconciled the previously incompatible objectives of seed palatability and nutritional density in *L. mutabilis*, establishing a paradigm shift in legume crop improvement. By demonstrating that protein biosynthesis and antioxidant accumulation remain phenotypically robust despite alkaloid minimization, we show that multiple nutritional dimensions can be simultaneously optimized without genetic trade-offs. The resulting next generation of cultivars integrating maximal protein content, elevated phenolic compounds (FRAP and ABTS), concentrated micronutrients profiles (Zn, Fe, Ca) and minimized alkaloids (<0.1%) position *L. mutabilis* as a transformative crop capable of addressing global malnutrition while advancing sustainable agro-industrial development. Strategic deployment of superior genotypes represents a critical contribution to global nutrition security and health equity.

## Figures and Tables

**Table 1:** Proximal composition of 12 *Lupinus* germplasm samples: protein, total fat, fiber, carbohydrates, ash and moisture content.

Germplasm	Protein (%)	Total Fat (%)	Fiber (%)	Carbohydrates (%)	Ash (%)	Moisture (%)
LmAnds16	44.89 ± 0.11 ^c^	18.86 ± 0.18 ^cde^	15.30 ± 0.27 ^abc^	14.94 ± 0.59 ^ef^	6.02 ± 0.03 ^a^	7.37 ± 0.13 ^c^
LmAnds77	42.81 ± 0.08 ^de^	18.53 ± 0.27 ^de^	13.08 ± 0.10 ^ef^	20.7 ± 0.16 ^c^	4.88 ± 0.07 ^b^	6.32 ± 0.19 ^d^
LmAnds167	41.59 ± 0.05 ^f^	18.87 ± 0.09 ^cde^	15.50 ± 0.15 ^ab^	19.18 ± 0.07 ^cd^	4.86 ± 0.05 ^b^	8.35 ± 0.16 ^ab^
LmFR11	43.19 ± 0.02 ^d^	19.77 ± 0.09 ^cd^	14.37 ± 0.39 ^bcd^	19.55 ± 0.26 ^cd^	3.13 ± 0.02 ^e^	6.94 ± 0.09 ^cd^
LmFR11s67	45.86 ± 0.04 ^b^	21.4 ± 0.15 ^b^	14.68 ± 0.41 ^bcd^	13.79 ± 0.51 ^fg^	4.29 ± 0.01 ^c^	8.73 ± 0.01 ^ab^
LmFR9s43	42.13 ± 0.10 ^ef^	18.45 ± 0.21 ^e^	14.1 ± 0.12 ^cde^	20.34 ± 0.19 ^cd^	4.99 ± 0.03 ^b^	6.68 ± 0.02 ^d^
INIAP 450 Andino *	47.17 ± 0.06 ^a^	20.11 ± 0.38 ^c^	13.49 ± 0.35 ^def^	15.95 ± 0.84 ^e^	3.28 ± 0.05 ^e^	8.51 ± 0.09 ^ab^
INIAP 451 Guaranguito *	44.51 ± 0.22 ^c^	23.87 ± 0.21 ^a^	15.48 ± 0.11 ^ab^	12.13 ± 0.09 ^g^	4.02 ± 0.08 ^d^	6.55 ± 0.25 ^d^
LmProin17 **	46.27 ± 0.17 ^b^	18.32 ± 0.15 ^e^	12.62 ± 0.04 ^f^	18.41 ± 0.03 ^d^	4.39 ± 0.02 ^c^	6.70 ± 0.04 ^d^
LmProin19 **	40.25 ± 0.23 ^g^	16.03 ± 0.15 ^f^	12.56 ± 0.09 ^f^	27.19 ± 0.42 ^b^	3.98 ± 0.04 ^d^	6.73 ± 0.32 ^d^
Average *L. mutabilis*	44.74	19.91	14.16	17.04	4.16	7.60
**Controls**						
Lalb2742 (*L. albus*) ***	34.29 ± 0.15 ^h^	12.07 ± 0.17 ^g^	8.86 ± 0.08 ^g^	40.81 ± 0.26 ^a^	3.98 ± 0.02 ^d^	8.63 ± 0.03 ^ab^
Lang4318 (*L. angustifolius*) ***	29.58 ± 0.13 ^i^	8.07 ± 0.30 ^h^	16.19 ± 0.26 ^a^	42.22 ± 0.08 ^a^	3.95 ± 0.01 ^d^	9.21 ± 0.24 ^a^
**CV (%)**	0.40	1.74	2.30	2.34	1.11	2.05

a, b, c, d, e, f, g, h, i: Significant differences (*p* < 0.05) in plant materials from the germplasm bank and controls, using Tukey’s test at 0.05. Lm: improved germplasm *L. mutabilis*, commercial varieties *L. mutabilis* (*), germplasm introduced *L. mutabilis* from Bolivia (**), control (***).

**Table 2:** Fatty acids profile of 12 samples of germplasm of *Lupinus*: saturated, monounsaturated and polyunsaturates fatty acids.

Plant Material	Saturated (SFA)			Monounsaturated (MUFA)		Polyunsaturated (PUFA)
	Palmitic Acid C16:0	Stearic Acid C18:0	Behenic Acid C22:0	Oleic Acid C18:1n9C	Linoleic Acid C18:2n6C	Linolenic Acid C18:3n3C
**Germplasm**						
LmAnds16	12.57 ± 0.03 ^a^	8.81 ± 0.01 ^b^	0.99 ± 0.05 ^d^	52.09 ± 0.01 ^e^	23.51 ± 0.04 ^e^	1.13 ± 0.01 ^j^
LmAnds77	11.48 ± 0.05 ^b^	9.91 ± 0.01 ^a^	1.19 ± 0.01 ^c^	57.2 ± 0.08 ^c^	16.38 ± 0.02 ^h^	1.65 ± 0.01 ^i^
LmAnds167	10.34 ± 0.02 ^cd^	4.45 ± 0.02 ^h^	0.72 ± 0.03 ^f^	49.85 ± 0.02 ^f^	31.63 ± 0.05 ^b^	2.52 ± 0.02 ^fg^
LmFR11	10.55 ± 0.08 ^c^	6.01 ± 0.09 ^e^	0.74 ± 0.07 ^f^	45.61 ± 0.01 ^h^	33.07 ± 0.02 ^a^	2.70 ± 0.01 ^ef^
LmFR11s67	8.46 ± 0.01 ^e^	9.75 ± 0.11 ^a^	0.88 ± 0.01 ^e^	59.26 ± 0.06 ^b^	18.53 ± 0.03 ^g^	2.22 ± 0.02 ^h^
LmFR9s43	11.21 ± 0.04 ^b^	5.57 ± 0.05 ^f^	0.76 ± 0.01 ^f^	53.51 ± 0.24 ^d^	25.01 ± 0.37 ^d^	3.36 ± 0.02 ^c^
INIAP 450 Andino *	10.46 ± 0.06 ^cd^	7.39 ± 0.01 ^c^	0.89 ± 0.01 ^e^	45.33 ± 0.05 ^h^	31.71 ± 0.01 ^b^	2.74 ± 0.05 ^e^
INIAP 451 Guaranguito *	10.28 ± 0.03 ^cd^	6.98 ± 0.11 ^d^	0.75 ± 0.00 ^f^	48.7 ± 0.02 ^g^	29.16 ± 0.08 ^c^	2.74 ± 0.04 ^e^
LmProin17 **	11.11 ± 0.12 ^b^	5.04 ± 0.02 ^g^	0.76 ± 0.01 ^f^	53.28 ± 0.10 ^d^	25.50 ± 0.06 ^d^	3.04 ± 0.02 ^d^
LmProin19 **	10.16 ± 0.01 ^d^	4.35 ± 0.03 ^h^	0.05 ± 0.01 ^f^	59.38 ± 0.25 ^b^	21.76 ± 0.33 ^f^	3.07 ± 0.02 ^d^
Average *L. mutabilis*	10.66	6.83	0.77	52.42	24.63	2.55
**Controls**						
Lalb2742 (*L. albus*) ***	8.05 ± 0.08 ^f^	2.37 ± 0.02 ^i^	3.03 ± 0.03 ^a^	66.7 ± 0.16 ^a^	11.2 ± 0.08 ^i^	4.26 ± 0.12 ^b^
Lang4318 (*L. angustifolius*) ***	12.31 ± 0.13 ^a^	7.07 ± 0.03 ^d^	1.77 ± 0.02 ^b^	38.34 ± 0.01 ^i^	32.29 ± 0.03 ^ab^	6.00 ± 0.02 ^a^
**CV (%)**	0.87	1.12	1.84	0.32	0.83	1.65

a, b, c, d, e, f, g, h, i, j: Significant differences (*p* < 0.05) in plant materials from the germplasm bank and controls, using Tukey’s test at 0.05. Lm: improved germplasm *L. mutabilis*, commercial varieties *L. mutabilis* (*), germplasm introduced *L. mutabilis* from Bolivia (**), control (***).

**Table 3:** Mineral contents of 12 samples of germplasm of Lupinus: macroelements (Ca, Mg, Na, K y P) and microelements (Cu, Fe, Mn y Zn).

Germplasm	Macroelements (g∙kg^−1^)					Microelements (ppm)			
	Ca	Mg	Na	K	P	Cu	Fe	Mn	Zn
LmAnds16	1.02 ± 0.01 ^fg^	2.02 ± 0.01 ^efg^	0.32 ± 0.01 ^b^	24.48 ± 0.24 ^a^	5.68 ± 0.15 ^b^	6.19 ± 0.19 ^cd^	49.03 ± 0.16 ^b^	23.99 ± 0.58 ^d^	50.42 ± 0.78 ^c^
LmAnds77	1.23 ± 0.05 ^e^	2.07 ± 0.01 ^def^	0.06 ± 0.01 ^i^	19.00 ± 0.23 ^b^	5.39 ± 0.14 ^bc^	7.13 ± 0.09 ^bcd^	49.66 ± 0.17 ^b^	33.56 ± 0.38 ^b^	45.17 ± 0.74 ^de^
LmAnds167	0.83 ± 0.01 ^i^	2.29 ± 0.01 ^bc^	0.22 ± 0.07 ^e^	18.49 ± 0.61 ^b^	5.98 ± 0.11 ^b^	6.69 ± 0.20 ^bcd^	39.68 ± 0.05 ^c^	23.02 ± 0.12 ^d^	42.26 ± 0.69 ^ef^
LmFR11	0.96 ± 0.02 ^fgh^	1.85 ± 0.05 ^g^	0.25 ± 0.01 ^d^	10.54 ± 0.28 ^d^	2.99 ± 0.09 ^f^	6.87 ± 0.24 ^bcd^	49.34 ± 1.88 ^b^	29.89 ± 0.32 ^bc^	40.24 ± 0.03 ^f^
LmFR11s67	0.87 ± 0.01 ^hi^	2.01 ± 0.01 ^fg^	0.15 ± 0.05 ^gh^	13.07 ± 0.17 ^c^	5.61 ± 0.03 ^b^	6.70 ± 0.19 ^bcd^	49.79 ± 0.23 ^b^	28.48 ± 0.16 ^c^	46.48 ± 0.19 ^d^
LmFR9s43	1.35 ± 0.03 ^d^	1.97 ± 0.06 ^fg^	0.34 ± 0.08 ^a^	13.07 ± 0.29 ^c^	4.06 ± 0.09 ^e^	4.03 ± 0.04 ^f^	33.87 ± 0.16 ^c^	20.14 ± 0.58 ^d^	39.91 ± 0.51 ^f^
INIAP 450 Andino *	0.91 ± 0.02 ^ghi^	2.20 ± 0.01 ^cde^	0.16 ± 0.09 ^fg^	7.97 ± 0.24 ^e^	4.78 ± 0.11 ^cd^	7.24 ± 0.13 ^abc^	47.20 ± 0.35 ^b^	28.55 ± 0.02 ^c^	40.46 ± 0.22 ^f^
INIAP 451 Guaranguito *	0.86 ± 0.03 ^hi^	2.40 ± 0.02 ^ab^	0.07 ± 0.01 ^i^	10.53 ± 0.05 ^d^	5.96 ± 0.17 ^b^	5.98 ± 0.15 ^de^	59.78 ± 0.29 ^a^	21.98 ± 0.25 ^d^	60.81 ± 0.53 ^a^
LmProin17 **	1.05 ± 0.01 ^f^	2.22 ± 0.01 ^bcd^	0.18 ± 0.09 ^f^	13.74 ± 0.39 ^c^	4.11 ± 0.02 ^de^	7.56 ± 0.30 ^ab^	46.15 ± 0.37 ^b^	29.97 ± 0.89 ^bc^	41.07 ± 0.70 ^f^
LmProin19 **	1.66 ± 0.05 ^c^	2.51 ± 0.09 ^a^	0.34 ± 0.01 ^a^	13.81 ± 0.22 ^c^	8.20 ± 0.21 ^a^	8.47 ± 0.42 ^a^	47.7 ± 2.74 ^b^	33.77 ± 0.23 ^b^	44.89 ± 1.89 ^de^
Average *L. mutabilis*	1.29	2.02	0.20	13.22	5.13	6.85	49.66	27.87	45.86
**Controls**									
Lalb2742 (*L. albus*) ***	2.04 ± 0.03 ^b^	1.86 ± 0.01 ^g^	0.28 ± 0.01 ^c^	9.69 ± 0.03 ^d^	4.20 ± 0.09 ^de^	4.07 ± 0.03 ^f^	37.23 ± 0.19 ^c^	142.11 ± 2.34 ^a^	56.16 ± 0.31 ^b^
Lang4318 (*L. angustifolius*) ***	2.91 ± 0.01 ^a^	2.22 ± 0.01 ^bcd^	0.13 ± 0.08 ^h^	10.23 ± 0.26 ^d^	4.47 ± 0.10 ^de^	4.87 ± 0.20 ^ef^	62.24 ± 0.06 ^a^	20.64 ± 0.20 ^d^	50.88 ± 0.35 ^c^
**CV (%)**	2.10	2.18	2.72	2.56	3.44	4.85	3.04	2.80	2.04

a, b, c, d, e, f, g, h, i: Significant differences (*p* < 0.05) in plant materials from the germplasm bank and controls, using Tukey’s test at 0.05. Lm: improved germplasm *L. mutabilis*, commercial varieties *L. mutabilis* (*), germplasm introduced *L. mutabilis* from Bolivia (**), control (***).

**Table 4:** Total antioxidants and antioxidant activity of 12 *Lupinus* germplasm samples produced in the Andean region of Ecuador.

Germplasm	Total Polyphenols (mg GAE∙g^−1^)	Total Flavonoids (mg CE∙g^−1^)	FRAP (µmol TE∙g^−1^)	ABTS (µmol TE∙g^−1^)
LmAnds16	6.23 ± 0.01 ^g^	0.66 ± 0.01 ^cd^	19.37 ± 0.45 ^de^	287.73 ± 15.47 ^de^
LmAnds77	5.68 ± 0.01 ^hi^	0.60 ± 0.02 ^def^	15.7 ± 0.81 ^fg^	236.56 ± 5.76 ^ef^
LmAnds167	10.46 ± 0.11 ^e^	0.73 ± 0.01 ^bc^	22.09 ± 0.96 ^cd^	352.87 ± 14.12 ^bc^
LmFR11	13.11 ± 0.06 ^c^	0.77 ± 0.01 ^ab^	25.73 ± 0.68 ^ab^	393.96 ± 11.52 ^ab^
LmFR11s67	12.24 ± 0.01 ^d^	0.76 ± 0.01 ^ab^	24.08 ± 0.47 ^bc^	382.51 ± 9.06 ^ab^
LmFR9s43	6.97 ± 0.05 ^f^	0.69 ± 0.01 ^bcd^	20.56 ± 0.07 ^d^	315.19 ± 7.55 ^cd^
INIAP 450 Andino *	15.61 ± 0.07 ^a^	0.84 ± 0.02 ^a^	28.39 ± 0.45 ^a^	432.98 ± 15.3 ^a^
INIAP 451 Guaranguito *	14.04 ± 0.12 ^b^	0.78 ± 0.01 ^ab^	26.53 ± 0.35 ^ab^	404.36 ± 11.02 ^ab^
LmProin17 **	5.88 ± 0.05 ^h^	0.62 ± 0.03 ^de^	17.3 ± 0.48 ^ef^	264.7 ± 7.83 ^def^
LmProin19 **	5.54 ± 0.09 ^i^	0.55 ± 0.01 ^efg^	13.8 ± 0.57 ^gh^	212.47 ± 9.43 ^f^
Average *L. mutabilis*	8.84	0.67	19.94	300.30
**Controls**				
Lalb2742 (*L. albus*) ***	2.2 ± 0.03 ^k^	0.46 ± 0.01 ^g^	11.43 ± 0.42 ^h^	38.95 ± 0.64 ^g^
Lang4318 (*L. angustifolius*) ***	3.11 ± 0.03 ^j^	0.51 ± 0.01 ^fg^	12.69 ± 0.26 ^gh^	51.48 ± 0.76 ^g^
**CV (%)**	0.942	3.377	3.947	4.832

a, b, c, d, e, f, g, h, i, j, k: Significant differences (*p* < 0.05) in plant materials from the germplasm bank and controls, using Tukey’s test at 0.05. Lm: improved germplasm *L. mutabilis*, commercial varieties *L. mutabilis* (*), germplasm introduced *L. mutabilis* from Bolivia (**), control (***).

**Table 5:** Alkaloid content of 12 *Lupinus* germplasm samples produced in the Andean region of Ecuador.

Germplasm	Alkaloids (%)
LmAnds16	0.04 ± 0.01 ^d^
LmAnds77	0.23 ± 0.03 ^d^
LmAnds167	0.63 ± 0.02 ^c^
LmFR11	1.15 ± 0.01 ^b^
LmFR11s67	0.72 ± 0.04 ^c^
LmFR9s43	0.04 ± 0.02 ^d^
INIAP 450 Andino *	1.77 ± 0.14 ^a^
INIAP 451 Guaranguito *	1.55 ± 0.05 ^a^
LmProin17 **	0.14 ± 0.02 ^d^
LmProin19 **	0.1 ± 0.02 ^d^
Average *L. mutabilis*	0.63
**Control**	
Lalb2742 (*L. albus*) ***	0.06 ± 0.02 ^d^
Lang4318 (*L. angustifolius*) ***	0.1 ± 0.02 ^d^
**CV (%)**	11.48

a, b, c, d: Significant differences (*p* < 0.05) in plant materials from the germplasm bank and controls, using Tukey’s test at 0.05. Lm: improved germplasm *L. mutabilis*, commercial varieties *L. mutabilis* (*), germplasm introduced *L. mutabilis* from Bolivia (**), control (***).

**Table 6:** Selected materials from the INIAP Lupinus germplasm bank and introduced materials.

Species	Name	Origin	Description of Germplasm
*L. mutabilis* (GM)	LmAnds16	Ecuador	Lines generated at INIAP from crosses between low-alkaloid genotypes (0.04%) and high-alkaloid genotypes (1.5–2%) [11].
	LmAnds77	Ecuador	
	LmAnds167	Ecuador	
	LmFR11	Ecuador	
	LmFR11s67	Ecuador	
	LmFR9s43	Ecuador	
*L. mutabilis* (VC)	INIAP 450 Andino *	Ecuador	Improved commercial variety developed in Ecuador through the introduction and selection method [12,13].
	INIAP 451 Guaranguito *	Ecuador	
*L. mutabilis* (GI)	LmProin17 **	Bolivia	Germplasm donated to INIAP by the PROINPA Foundation of Bolivia in 2021. The initial material was genetically mixed and purified through three consecutive years of selection. This material is characterized by its low alkaloid content [11].
	LmProin19 **	Bolivia	
*L. albus* (GI)	Lalb2742 ***	NA	Accession ECU-2742 from the INIAP germplasm bank [11].
*L. angustifolius* (GI)	Lang4318 ***	NA	Accession ECU-4318 from the INIAP germplasm bank [11].

VC: commercial variety (*), GM: improved germplasm, GI: introduced germplasm (**), control (***).

**Table 7:** Results of soil analysis for pH and macro- and micronutrient content of the plot where the *Lupinus germplasm* was planted.

Edaphoclimatic Conditions at Planting	
**Experimental site**	
Location	Cutuglahua, Pichincha, Ecuador
Latitude	00°22′05″ S
Longitude	78°33′31″ W
Altitude	3050 m above sea level
Planting season	January to September 2022
Average relative humidity	82%
Average minimum temperature	3.5 °C
Average maximum temperature	20.6 °C
Precipitation	1084 mm
**Soil analysis**	
pH	5.63
Nitrogen (N)	60 ppm
Phosphorus (P)	142 ppm
Sulfur (S)	23 ppm
Boron (B)	0.38 ppm
Zinc (Zn)	6 ppm
Copper (Cu)	13.8 ppm
Iron (Fe)	424 ppm
Manganese (Mn)	10.7 ppm
Potassium (K)	0.05 meq/100 g
Calcium (Ca)	3.35 meq/100 g
Magnesium (Mg)	0.15 meq/100 g
Organic Matter	8.4%

Source: Rodriguez et al. [14].

## Data Availability

The original contributions presented in this study are included in the article. Further inquiries can be directed at the corresponding author.
